# Detection of urinary miRNAs for diagnosis of clear cell renal cell carcinoma

**DOI:** 10.1038/s41598-020-77774-9

**Published:** 2020-12-04

**Authors:** Giovanni Cochetti, Luigi Cari, Giuseppe Nocentini, Vincenza Maulà, Chiara Suvieri, Rosy Cagnani, Jacopo Adolfo Rossi De Vermandois, Ettore Mearini

**Affiliations:** 1 Urology Clinic, Department of Medicine and Surgery, University of Perugia, Perugia, Italy; 2 Pharmacology Section, Department of Medicine and Surgery, University of Perugia, Perugia, Italy

**Keywords:** Cancer, Biomarkers, Urology

## Abstract

The lack of symptoms at the early stages of clear cell renal cell carcinoma (ccRCC) allows the tumour to metastasize, leading to a dramatic reduction in patient survival. Therefore, we studied and set up a method based on urinary microRNAs (miRNAs) for the diagnosis of ccRCC. First, miRNA expression in ccRCC specimens and kidney tissues from healthy subjects (HSs) was investigated through analysis of data banks and validated by comparing expression of miRNAs in ccRCC and adjacent non-cancerous kidney tissue specimens by RT-qPCR. Subsequently, we developed an algorithm to establish which miRNAs are more likely to be found in the urine of ccRCC patients that indicated miR-122, miR-1271, and miR-15b as potential interesting markers. The evaluation of their levels and three internal controls in the urine of 13 patients and 14 HSs resulted in the development of a score (7p-urinary score) to evaluate the presence of ccRCC in patients. The resulting area under the Receiver Operating Characteristic (ROC) curve, sensitivity, and specificity were equal to 0.96, 100% (95% CI 75–100%), and 86% (95% CI 57–98%), respectively. In conclusion, our study provides a proof of concept that combining the expression values of some urinary miRNAs might be useful in the diagnosis of ccRCC.

## Introduction

In 2018, 400,000 people were diagnosed in the world with renal cell carcinoma (RCC), of which 65–70% had clear cell RCC (ccRCC) as a prevalent histological type^1^ and 175,000 people died from the disease^2^. The lack of symptoms at the early stages allows the tumour to grow and metastasize, leading to a dramatic reduction in patient survival^3^. Indeed, the risk of metastasis is less than 5% for a tumour diameter below 3 cm, 18% for a tumour diameter of 6–7 cm, and up to 28% in a more advanced disease^4,5^. These data indicate the necessity to diagnose RCC as early as possible. Many studies have suggested the use of imaging techniques such as a CT scan and MRI, while others have started focusing on molecular diagnostic techniques with an improved cost–benefit profile^6^.

MicroRNAs (miRNAs) are small noncoding RNAs of 18–22 nucleotides, which not only inhibit RNA translation and promote RNA degradation but also regulate the transcription and splicing processes^7,8^. Not surprisingly, miRNAs are implicated in the development and progression of numerous tumours^9,10^, including ccRCC^11–14^. MicroRNAs are stable and present in bodily fluids (e.g., blood plasma, urine, saliva, and semen) within microvesicles, apoptotic bodies, or membrane-free carriers^8,14^, which make these RNAs promising biomarkers for acute diseases^15^ and cancer diagnosis and prognosis^10,16^. Although many studies have evaluated miRNA expression in tissues and serum of patients with ccRCC, only a few have investigated the potential role of miRNAs as urinary biomarkers^17–21^.

Our study was aimed to develop a method for diagnosis of ccRCC based on the measurement of urinary miRNAs. We demonstrated that combining the expression values of three urinary miRNAs (miR-122, miR-1271, and miR-15b) and internal controls may be useful in the diagnosis of ccRCC.

## Results

### Evaluation of miRNA overexpression in ccRCC through data bank analysis and laboratory data

**Figure 1:**
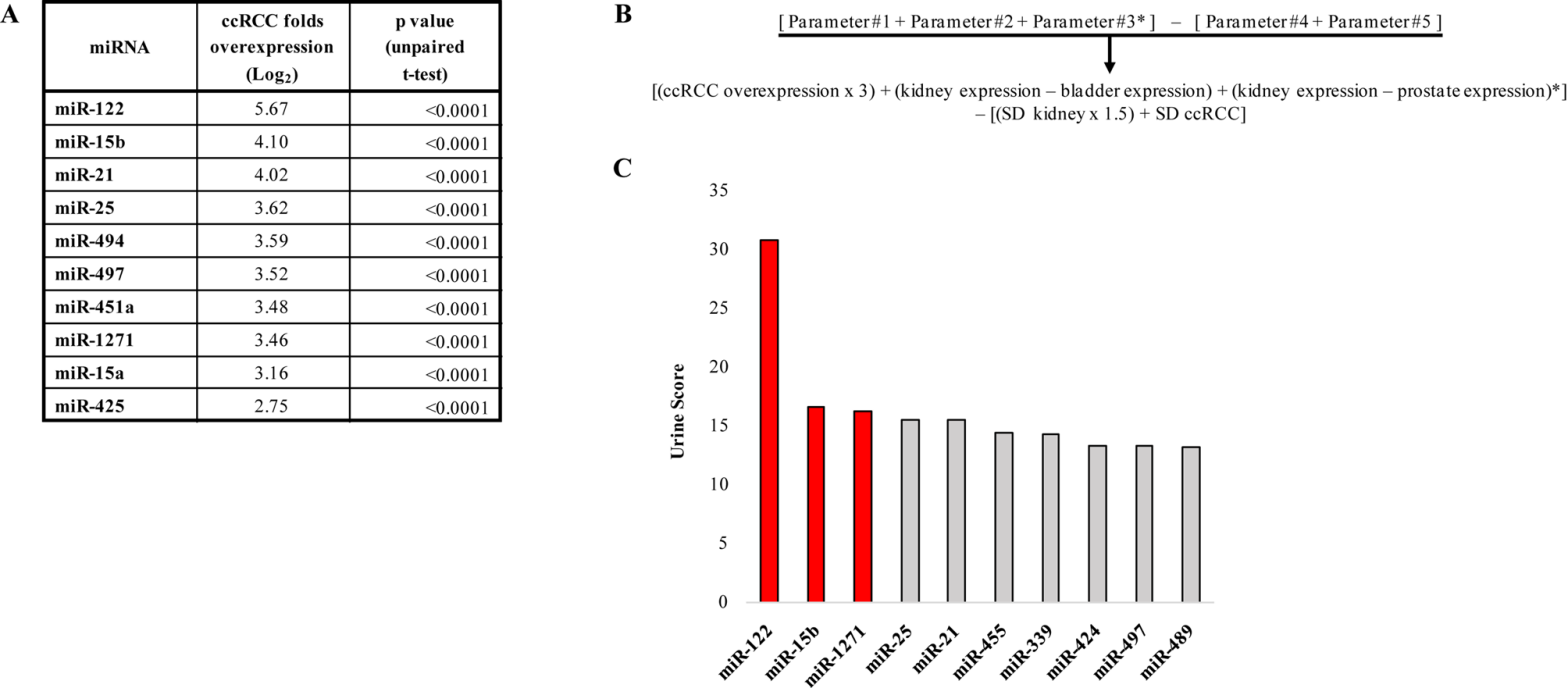
The best urinary miRNA markers as derived by data bank analysis and algorithm. (**A**) Top 10 miRNAs overexpressed in ccRCC samples compared with their expression in kidney samples from healthy subjects (HSs), based on data bank analysis. (**B**) Algorithm used to calculate the probability for a miRNA to represent a urinary marker of ccRCC. Overexpression in ccRCC, expression in the kidney, bladder, and prostate (the latter used to calculate the score in men only *), and standard deviation (SD) of miRNA expression in normal tissue and ccRCC were calculated using the data bank values expressed as Log_2_. **C**, The top 10 miRNAs and their scores potentially representing the best urinary markers of ccRCC, as suggested by the algorithm.

To identify the best miRNA candidates for urinary biomarkers of ccRCC, we first analyzed miRNA expression in data banks to find miRNAs that appear to be overexpressed and underexpressed in ccRCC specimens compared with their expression in kidney specimens from healthy subjects (HSs). Twenty-seven miRNAs were significantly overexpressed, at least 3.3-folds (1.73 Log_2_ folds), in the ccRCC specimens, and 30 miRNAs were significantly underexpressed, at least 1.7-folds (0.77 Log_2_ folds) in the ccRCC specimens (Fig. 1A and Supplementary Table S1).

To validate bioinformatics data, we tested expression levels of eight miRNAs deemed overexpressed/underexpressed in ccRCC versus kidney specimens from HSs. In particular, we tested the two miRNAs resulting the most overexpressed (miR-122 and miR-15b), four more miRNA, randomly chosen among the overexpressed miRNAs (miR-1271, miR-629, miR-625, and miR-93), the miRNA resulting the most underexpressed (miR-1260a) and another randomly chosen underexpressed miRNA (miR-369). The expression levels of miRNAs, normalized to that of the reference gene RNA, RNU6-2, were evaluated by quantitative reverse transcription PCR (RT-qPCR) in ccRCC and adjacent non-cancerous kidney tissue specimens from 17 patients. The Ct values of the tested miRNAs were considered reliable when the RNU6-2 Ct was less than 30. Three samples from three patients were not reliable; therefore, we obtained expression values for 14 ccRCC specimens and the corresponding adjacent non-cancerous kidney tissue samples (Table 1 and Supplementary Table S2). Among the miRNAs resulting overexpressed, miR-122 was found to be overexpressed in all but one cancer specimens, and four miRNAs were found to be overexpressed in more than 70% of the ccRCC specimens. The two miRNAs that were identified to be underexpressed in ccRCC, according to the bioinformatic analysis, were underexpressed in 57% of the ccRCC specimens (Supplementary Table S2).

**Table 1:** RNU6-2-normalized miRNA expression in ccRCC and correspondent adjacent non-cancerous kidney tissue (Log_2_ values). Experimental-derived data are compared to data derived from data bank analysis.

miRNA	Overexpression folds	Overexpression (positive values, Log_2_) of miRNAs by ccRCC				
		Experimental-derived data (ccRCC samples vs correspondent adjacent non-cancerous kidney tissue)				Bioinformatics-derived data (mean of ccRCC samples vs mean of kidney samples from HSs)
		Min value	Max value	Passed KS test *	p value (paired test)	Overexpression folds
miR-122	5.74	− 2.90	9.52	Yes	< 0.0001	5.67
miR-1271	0.32	− 1.99	2.49	No	0.3258	3.46
miR-15b	1.38	− 0.79	3.28	No	0.0031	4.10
miR-625	1.15	− 1.33	3.78	Yes	0.0180	2.04
miR-629	0.60	− 2.65	3.28	Yes	0.1993	2.39
miR-93	− 0.01	− 1.81	1.74	No	0.9515	1.82

miRNA	Underexpression folds	Underexpression (negative values, Log_2_) of miRNAs by ccRCC				
		Experimental-derived data (ccRCC samples vs correspondent adjacent non-cancerous kidney tissue)				Bioinformatics-derived data (mean of ccRCC samples vs mean of kidney samples from HSs)
		Min value	Max value	Passed KS test *	p value (paired test)	Underexpression folds
miR-1260a	− 0.12	− 4.69	3.55	No	0.9878	1.83
miR-369	− 0.82	− 7.29	5.01	Yes	0.4158	0.77

* If Kolmogorov–Smirnov (KS) test (a normality test) was passed, the paired t-test was used to calculate p value, otherwise the Wilcoxon matched-pairs signed-rank test was used.

The differences in their levels of expression between the ccRCC and adjacent non-cancerous kidney tissue specimens were significant for three miRNAs, and the mean levels of overexpression/underexpression found with data bank analysis were substantially confirmed by our experimental data for three miRNAs (Table 1). Despite the high inter-patient variability and a relatively low number of the patients evaluated, the experimental data confirmed the mean variation trend derived from bioinformatics (overexpressed/underexpressed in cancer) for seven of the eight miRNAs. Therefore, we concluded that the experimental data substantially confirmed the data bank analysis.

### Development of an algorithm to find urinary miRNAs with the highest probability of becoming ccRCC biomarkers

To find among the overexpressed genes the miRNAs that could potentially be used as urinary markers for ccRCC, we developed an algorithm, considering confounding factors and the variability of miRNA expression in various specimens as reported in detail below. The final score produced by the algorithm was derived from a sum of five scores, based on five parameters.

Parameter #1 considered the level of miRNA overexpression in ccRCC specimens compared with its expression in HS specimens. The value (expressed as Log_2_) was multiplied by 3. Parameters #2 and #3 were considered to take into account the confounding factors (urinary miRNAs are derived not only from the kidney but also from the urinary ducts, bladder, and prostate in men^22^). We reasoned that if a miRNA was expressed by the kidney and at similar or even higher levels by urinary ducts, bladder, and prostate (determining a background noise in the urine) was harder to detect its ccRCC-dependent increase in the urine. In other words, parameters #2 and #3 take into account the background noise of miRNAs derived from tissues/organs different from the kidney, because the lower it was, the higher the possibility that the miRNA represented a marker of ccRCC. In particular, our algorithm accounted for the difference between the miRNA expression in the kidney and bladder using parameter #2, which was positive if miRNA expression was higher in the kidney than in the bladder and negative otherwise. When applying the algorithm to a male, the difference between the miRNA expression in the kidney and prostate was also considered (parameter #3). Another crucial parameter for a miRNA candidate to be a urinary marker of ccRCC was the difference in the expression of the miRNA in the kidneys from different subjects and ccRCC from different patients. High standard deviation (SD) values of the miRNA expression in the kidney from HSs and ccRCC would lower the possibility of finding a miRNA whose range of expression in ccRCC did not overlap with that in a healthy kidney.

For this reason, in our algorithm, the value resulting from parameters #1, #2, and #3 was decreased by SD values of the miRNA expression in the kidney from HSs (parameter #4) and ccRCC (parameter #5). The kidney SD was considered to be more relevant than that of ccRCC. The algorithm used to select the best candidates for the diagnosis of ccRCC through the evaluation of the above-mentioned parameters is summarized in Fig. 1B. The score of each parameter was obtained by using the values derived from the Genevestigator v3 suite^23^. While we believe that the chosen parameters account for factors determining the usefulness of urinary miRNA as ccRCC markers, the algorithm suffers from at least two limitations. First, the algorithm does not include a parameter accounting for the tendency of the miRNAs to be secreted by ccRCC. If the tendency had been equal, the predictivity of the algorithm would have been high. Since the tendency of the miRNAs to be secreted by ccRCC is unknown, it easy to predict that the miRNAs selected by the algorithm may represent or may not ccRCC urinary markers. The second limitation of the algorithm is that the factors by which the parameter values were multiplied (Supplementary Table S3) were chosen by us to give different weights to the different parameters but are arbitrary in their values. However, we chose the factors so that they represent the different relevance of the parameters (e.g., overexpression value is more important than the SD of the miRNA expression by ccRCC), weighting the chosen parameters.

Figure 1C and Supplementary Figure S1 show the algorithm-derived urinary scores for the 27 miRNAs overexpressed in ccRCC at least 3.3-folds, and Supplementary Table S3 shows values determining the final urine score. miR-122 was found to be the best candidate for potential ccRCC urinary marker with a score equal to 30.7 and was selected for further studies. Twelve miRNAs showed a score between 10 and 17 with relatively small differences among their scores. We decided to select the two miRNAs with the highest score: miR-1271 and miR-15b.

### Urinary miR-122 and miR-1271 showed a good predictive power

To confirm the predictive power of the three miRNAs selected through the algorithm, their presence was tested in the urine of 14 HSs and 13 patients with ccRCC.

To examine whether there was enough RNA of good quality, the RT-qPCR of three internal controls (miR-16, miRTC, and cel-miR-39) was also performed for each sample. When RT-qPCR of a miRNA (whichever it was: investigated or internal control) of a sample showed a Ct value > 38, another RT-qPCR of the sample was performed. If both assays showed Ct values > 38, the miRNA was considered absent in the sample, and its Ct was set to 40 by convention.

Table 2 shows the mean expression of miRNAs in urine samples of patients with ccRCC as compared to that of HSs. When not normalized by internal controls, miRNAs resulted to be overexpressed in urine samples of patients with ccRCC. Supplementary Table S4 shows the mean Ct of miRNAs and internal controls. The mean Ct of miR-122 was lower in the urine of the patients than in the urine of the HSs, indicating that the mean level of miR-122 was 14.9-fold (3.9 Log_2_ folds) higher in the urine of the patients with ccRCC, confirming the indication derived from the algorithm. Although miR-1271 was expressed at similar levels in the ccRCC and adjacent non-cancerous kidney tissue specimens (see Table 1), a 3.3-fold (1.74 Log_2_ fold) higher amount of miR-1271 in the urine of the patients with ccRCC was observed, confirming the indication derived from the algorithm. Surprisingly, the amount of miR-15b was similar in the urine from the patients and HSs.

**Table 2:** Not normalized miRNA levels in the urine samples of ccRCC patients as compared to urine samples of healthy subjects (HSs) (Log_2_ values). Urinary miRNA levels are compared to the overexpression of RNU6-2-normalized miRNAs in ccRCC specimens as compared to the corresponding adjacent non-cancerous kidney tissue samples.

miRNA	INCREASED LEVELS (positive values, Log_2_) of miRNAs in the urine of ccRCC patients and OVEREXPRESSION of miRNAs (positive values, Log_2_) by ccRCC specimens			
	Urine (mean of ccRCC samples vs mean of HS samples)			Tissue (ccRCC samples vs correspondent adjacent non-cancerous kidney tissue, see Table 1)
	Folds	Passed KS test *	p value (unpaired test)	Folds (p values)
miR-122	3.90	No	0.0032	5.74 (< 0.0001)
miR-1271	1.74	Yes	0.0150	0.32 (0.3250)
miR-15b	0.86	Yes	0.4890	1.38 (0.0017)

*If Kolmogorov–Smirnov (KS) test (a normality test) was passed, the unpaired t-test was used to calculate p value, otherwise the Mann–Whitney test was used.

**Figure 2:**
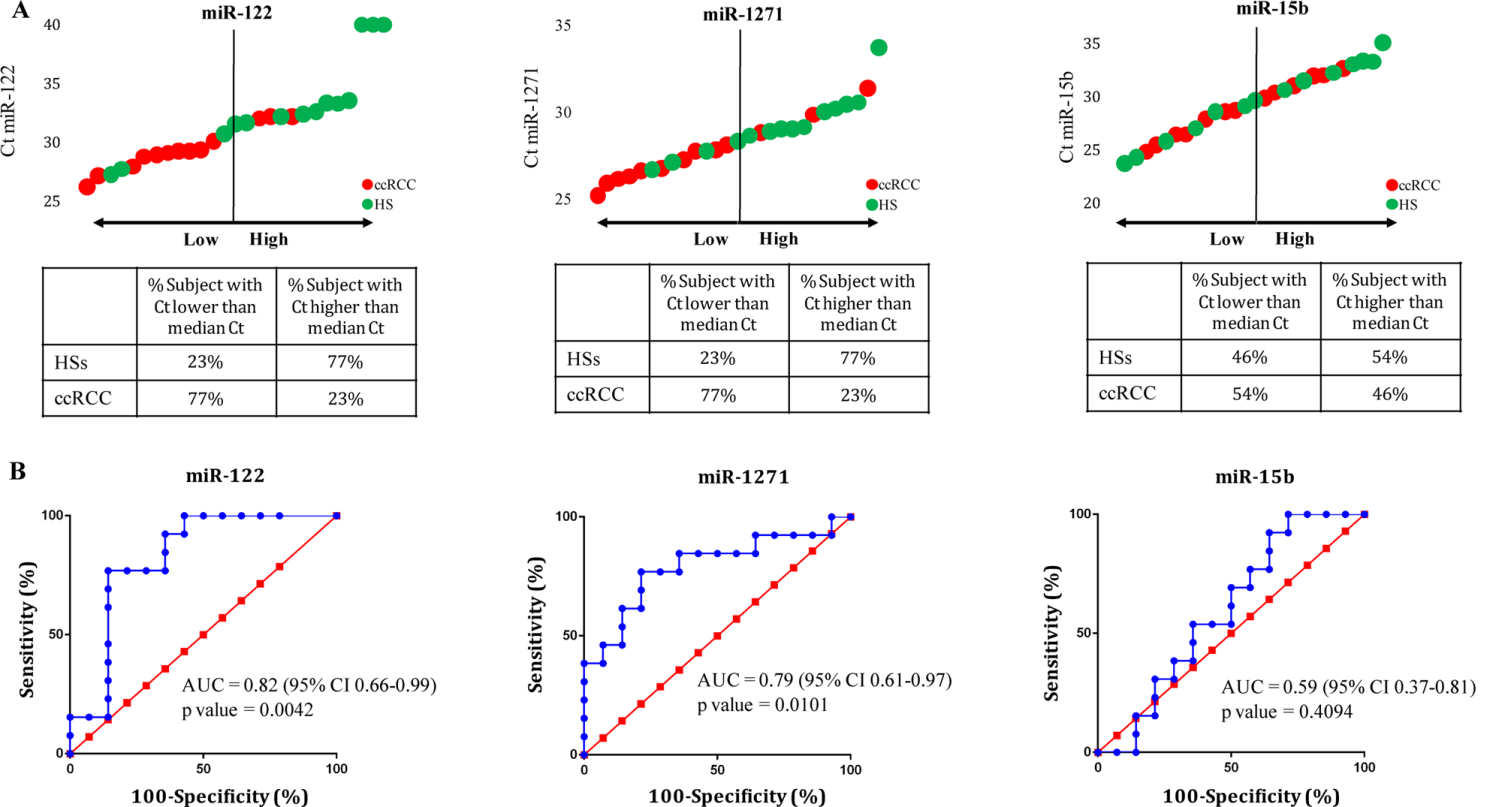
Expression levels of urinary miRNAs and ROC curves. (**A**) Ct value of miR-122, miR-1271, and miR-15b in the urine of HSs (green dots) and patients with ccRCC (red dots); in the Tables, the percentage of HSs and ccRCC patients with Ct value lower and higher than the median Ct is reported. Median Cts were equal to 31.46, 28.31, and 29.48 for miR-122, miR-1271, and miR-15b, respectively. (**B**) ROC curves of miR-122, miR-1271, and miR-15b.

To determine which miRNA has higher discriminating power for urine from patients with ccRCC and HSs, we analyzed the levels of the three miRNAs in the urine of each patient and HS. We calculated the median Ct value of each miRNA considering both ccRCC and HSs as a whole population. Figure 2A shows that urinary miR-122 and miR-1271 discriminated better ccRCC and HS samples than did miR-15b. Indeed, 77% of the Ct values of urinary miR-122 and miR-1271 were lower than the median Ct derived from patients with ccRCC, meaning that 77% of patients with ccRCC had the expression of these miRNAs higher than the median expression. Not surprisingly, miR-15b showed a much lower discrimination power.

As expected, the AUCs of miR-122 and miR-1271 were significant and interesting, showing a value of about 0.8 (Fig. 2B), comparable to the one obtained by other studies with other urinary miRNAs^19,20^. Supplementary Figure S2 and S3 show that following normalization with internal controls the AUCs of miR-122 and miR-1271 were lower, with the only exception of miRTC-normalized miR-1271 showing an AUC equal to 0.77. However, the values of ΔCt miR-1271/miRTC in ccRCC patients were higher than those of HSs and not lower as observed with miRNA-1271 alone (Fig. 2B). The same result was observed with miR-122/miRTC, suggesting that both miR-122 and miR-1271 appear overexpressed in the urine of patients with ccRCC when not normalized and expressed at the same level or even at lower levels when normalized by miRTC (observed with both miR-122 and miR-1271) or the other internal controls (observed only with miR-1271). The expression of miR-15b normalized by each internal control appears to be lower in the urine of patients with ccRCC (Supplementary Figure S4). If observed from the point of view of internal controls, mean Ct of miR-16 (the internal control considered to be a sort of housekeeping signal and by which it is evaluated both the quality of the urine sample and the quality of reactions) is similar in the urine of patients with ccRCC and HSs (Supplementary Table S4 and Supplementary Figure S5A), suggesting that sampling and handling of the urine of patients with ccRCC and HSs were similar and that miR-16 was present at similar levels in HSs and patients. On the contrary, mean Ct of cel-miR-39 and miRTC (the internal controls of RNA extraction and reverse transcriptase reaction) are different (about 4 and 6 Ct, p = 0.0595 and p = 0.0005, respectively) in the urine of patients with ccRCC and HSs (Supplementary Table S4 and Supplementary Figure S5B-C). Data may suggest that cel-miR-39 and miRTC have a different amplification rate in the urine of ccRCC as compared to HSs, for unknown reasons. Indeed, AUCs of cel-miR-39 and miRTC are equal to 0.66 (not significant) and 0.87 (significant), respectively (Supplementary Figure S6).

We also evaluated if there was a correlation between the levels of urinary miRNAs and miRNAs expressed by the corresponding ccRCC specimens (Supplementary Figure S7). We did not find any significant correlation, likely due to the low number of patients for which we had both ccRCC specimens and samples (12 patients). However, the tendency of a direct correlation was present, concerning miR-122 and miR-15b. The same tendency was observed with miR-1271 when two outliers were not considered (Supplementary Figure S7B). Data may confirm that the levels of urinary miRNAs depend on their expression by ccRCC.

In summary, two out of the three miRNAs indicated by the algorithm as potential urinary markers of ccRCC were confirmed to be overexpressed in the urine of several ccRCC patients. On the contrary, miR-15b was not confirmed to be overexpressed in the urine of ccRCC patients. Likely, the miR-15b derived from other tissues (e.g., bladder and prostate), its degradation rate, and a low tendency of miR-15b to diffuse in the urine are responsible for the data found with miR-15b, even if we cannot exclude that the data concerning urinary miR-15b are due to inter-patient and inter-HS variability and an unfortunate choice of patients.

Despite the prediction ability of urinary miR-122 and miR-1271 was good, it was still suboptimal, with both false-negative (FN) and false-positive (FP) rates of 23% (Fig. 2).

### Mathematical treatment of the data to obtain parameters that can be combined

Considering that two out of the three miRNAs had a good performance and that also internal controls showed a different Ct in patients and HSs (Supplementary Table S4), we tried to increase the discriminating power of data by combining them using an unsupervised approach.

As a first step, the urinary Ct (or ΔCt) values in HSs and patients with ccRCC were calculated for miR-122, miR-1271, and miR-15b, with and without normalization by internal controls, resulting in 12 parameters (Supplementary Table S5). Some mean values resulted in being lower in ccRCC than HSs (parameters 1–4, 6, 7), whereas other mean values resulted in being higher in ccRCC than HSs (parameters 5, 8–12).

After calculating the mean ranges (mean ± SD) of the urinary Ct (or ΔCt) in the patients with ccRCC, the urinary Ct values of the parameters with mean values in ccRCC lower than in HSs (parameters 1–4, 6, 7) were subtracted with the mean + SD values of urinary Ct or ΔCt values in the patients with ccRCC [(Ct or ΔCt) − (Ct or ΔCt mean + SD of ccRCC)]. When urinary ΔCt values of the parameters showed mean values in ccRCC higher than in HSs (parameters 5, 8–12), the mean-SD values of urinary ΔCt in the patients with ccRCC were subtracted with ΔCt [(ΔCt mean-SD of ccRCC) − (ΔCt)].

Table 3 shows that the frequency of the Ct and ΔCt derivatives with negative values (highlighted in bold and representing a diseased value) is higher among ccRCC than among HSs urine samples.

**Table 3:** Ct and ΔCt derivatives calculated using Ct and ΔCt values and the range of values observed in diseased patients, as reported in Supplementary Table S5.

Parameter		# 1	# 2	# 3	# 4	# 5	# 6	# 7	# 8	# 9	# 10	# 11	# 12
Parameter description		miR-122 Ct *	miR-1271 Ct *	miR-15b Ct *	miR-122/miR-16 ΔCt *	miR-122/miRTC ΔCt **	miR-122/cel-miR-39 ΔCt *	miR-1271/miR-16 ΔCt *	miR-1271/miRTC ΔCt **	miR-1271/cel-miR-39 ΔCt **	miR-15b/miR-16 ΔCt **	mi-15b/miRTC ΔCt **	miR-15b/cel-miR-39 ΔCt **
		Value ***											
Sample code ***	*ccRCC 2*	− **1.96**	− **1.48**	− **0.60**	− **2.85**	− **3.33**	− **0.75**	− **3.18**	− **4.35**	− **3.89**	− **4.21**	− **5.91**	− **6.14**
	*ccRCC 3*	− **2.39**	− **2.00**	*0.37*	− **3.41**	− **2.65**	− **2.05**	− **3.83**	− **3.58**	− **2.50**	− **5.05**	− **6.63**	− **6.24**
	*ccRCC 4*	− **2.03**	− **1.16**	− **2.86**	− **0.48**	− **7.09**	− **0.22**	− **0.42**	− **8.50**	− **4.81**	− **4.39**	− **7.48**	− **4.48**
	*ccRCC 5*	− **2.10**	− **2.63**	− **3.00**	*1.92*	− **3.52**	*0.57*	*0.58*	− **3.53**	− **4.20**	− **6.72**	− **3.84**	− **5.20**
	*ccRCC 6*	− **3.25**	− **4.03**	− **5.03**	− **6.87**	*2.14*	− **3.58**	− **8.46**	*2.38*	*0.20*	*2.95*	*2.70*	− **0.17**
	*ccRCC 7*	*0.82*	− **0.44**	− **1.72**	− **2.23**	− **4.49**	*1.66*	− **4.30**	− **3.77**	− **4.56**	− **0.93**	− **3.17**	− **4.65**
	*ccRCC 8*	− **5.08**	− **3.02**	− **6.18**	− **0.73**	− **0.56**	− **2.62**	*0.52*	− **3.16**	− **3.60**	− **3.87**	− **0.68**	− **1.81**
	*ccRCC 9*	− **4.15**	− **1.44**	− **3.69**	− **0.60**	− **1.60**	− **2.68**	*1.30*	− **4.85**	− **4.19**	− **5.56**	− **3.28**	− **3.31**
	*ccRCC 10*	− **2.41**	− **3.35**	− **5.06**	− **2.02**	− **3.62**	− **4.53**	− **3.77**	− **3.22**	*1.31*	− **1.03**	− **2.19**	*1.65*
	*ccRCC 11*	− **2.03**	*0.66*	*1.07*	− **5.13**	− **3.03**	− **5.93**	− **3.25**	− **6.26**	− **0.92**	− **3.67**	− **7.35**	− **2.70**
	*ccRCC 12*	− **1.24**	− **2.49**	− **6.66**	− **0.90**	− **7.25**	− **1.77**	− **2.96**	− **6.54**	− **1.14**	*0.62*	− **3.05**	*1.66*
	*ccRCC 13*	*0.87*	− **2.87**	− **1.08**	− **2.55**	*1.13*	− **1.67**	− **7.10**	*4.33*	*1.25*	− **1.20**	*1.86*	− **1.91**
	*ccRCC 17*	*0.90*	*2.11*	*0.54*	− **3.03**	− **1.73**	− **3.76**	− **2.63**	− **3.48**	− **1.61**	− **2.31**	− **2.59**	− **1.41**
	*HS 1*	*0.89*	*4.48*	*3.48*	− **5.50**	*6.17*	− **3.84**	− **2.72**	*2.04*	− **3.91**	− **2.79**	*2.36*	− **4.28**
	*HS 2*	*2.01*	− **1.47**	− **2.00**	− **1.64**	*0.55*	*4.80*	− **5.93**	*3.49*	− **5.48**	− **0.05**	*3.34*	− **6.32**
	*HS 3*	*0.36*	− **0.92**	− **0.10**	− **1.64**	*2.73*	− **1.65**	− **3.73**	*3.47*	− **1.23**	− **3.60**	*1.97*	− **3.42**
	*HS 4*	*1.11*	− **0.38**	*1.79*	− **4.34**	*2.31*	− **5.93**	− **6.64**	*3.26*	*3.26*	− **2.04**	*0.41*	− **0.28**
	*HS 5*	− **3.47**	− **0.23**	− **5.80**	*0.91*	*0.47*	− **0.27**	*3.34*	− **3.31**	− **7.13**	− **4.28**	*1.58*	− **2.93**
	*HS 6*	*1.33*	− **0.04**	*0.77*	− **2.74**	*5.69*	− **1.71**	− **4.92**	*6.52*	− **1.08**	− **2.40**	*5.03*	− **3.26**
	*HS 7*	− **4.06**	− **2.54**	− **7.33**	*0.75*	− **1.57**	− **1.60**	*1.46*	− **3.63**	− **4.08**	− **3.18**	*0.48*	− **0.66**
	*HS 8*	*8.81*	*1.03*	*1.76*	*1.64*	− **3.12**	− **0.31**	− **6.95**	*4.12*	*3.93*	− **0.29**	*2.71*	*1.83*
	*HS 9*	*0.27*	*1.21*	*1.57*	− **0.68**	− **0.80**	− **7.32**	− **0.55**	− **2.28**	*2.22*	− **6.32**	− **3.32**	*0.49*
	*HS 10*	*8.81*	− **2.11**	− **7.92**	*13.50*	− **13.64**	*11.52*	*1.77*	− **3.26**	− **4.76**	− **2.47**	*1.87*	− **0.32**
	*HS 11*	*8.81*	− **0.54**	− **0.86**	*8.67*	− **2.90**	− **12.34**	− **1.49**	*5.91*	*17.53*	− **4.70**	*5.55*	*16.48*
	*HS 12*	*2.23*	− **0.20**	− **2.39**	− **0.95**	− **5.65**	− **0.35**	− **4.19**	− **3.76**	− **1.38**	− **0.13**	− **2.25**	− **0.56**
	*HS 13*	*2.11*	*0.79*	− **3.04**	− **2.01**	− **0.23**	− **5.95**	− **4.14**	*0.55*	*3.11*	*1.46*	*3.70*	*5.57*
	*HS 14*	− **0.55**	*1.38*	− **4.46**	− **1.37**	− **2.56**	− **3.01**	− **0.25**	− **5.03**	− **3.08**	− **0.42**	*0.13*	*1.39*

*Ct (or ΔCt)—mean + SD of ccRCC.

**Ct mean-SD of ccRCC—Ct (or ΔCt).

***In italic the positive values which are in the healthy range; in bold the negative values which are in the diseased range.

### Increase of the predicting power of the test by the combination of parameters

**Figure 3:**
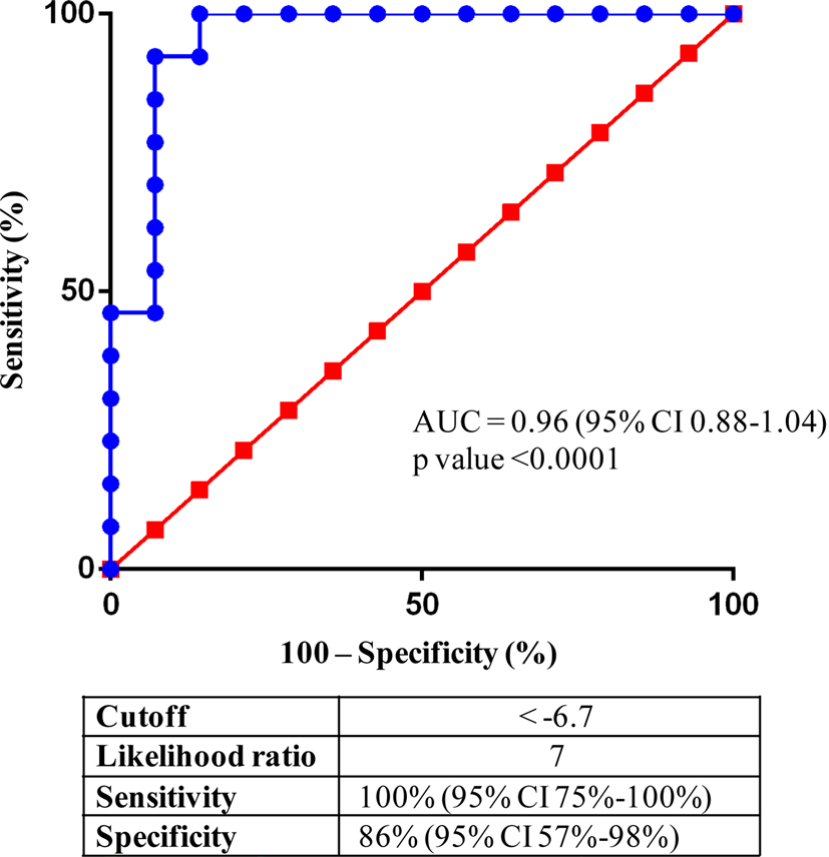
Predictive power derived from parameter combination (7p-urinary score). ROC curve based on the sum of parameters #2, #4, #5, #7, #8, #11, and #12 as shown in Table 4.

To obtain high predictive power from the data, for each HS and patient, we calculated the values derived from the sum of N parameters (6 < N < 12) and prepared the corresponding ROC curves. The sum of parameters #2, #4, #5, #7, #8, #11, and #12 (7p-urinary score) gave the best result (Table 4), and the correspondent ROC curve showed an AUC of 0.96 and p < 0.0001 (Fig. 3). Moreover, at a cutoff of − 6.7 (meaning that values lower than -6.7 indicate the presence of cancer), the 7p-urinary score showed a sensitivity of 100% and a specificity of 86%.

**Table 4:** Sum of the Ct (or ΔCt) value derivatives of seven parameters (#2, #4, #5, #7, #8, #11, and #12) as defined in Table 3.

Parameter		# 2	# 4	# 5	# 7	# 8	# 11	# 12	Parameter Sum *	Highest value
Parameter description		miR-1271 Ct	miR-122/miR-16 ΔCt	miR-122/miRTC ΔCt	miR-1271/miR-16 ΔCt	miR-1271/miRTC ΔCt	miR-15b/miRTC ΔCt	miR-15b/cel-miR-39 ΔCt		
Sample code **	*ccRCC 2*	− **1.48**	− **2.85**	− **3.33**	− **3.18**	− **4.35**	− **5.91**	− **6.14**	− 27.24	− 7.11
	*ccRCC 3*	− **2.00**	− **3.41**	− **2.65**	− **3.83**	− **3.58**	− **6.63**	− **6.24**	− 28.34	
	*ccRCC 4*	− **1.16**	− **0.48**	− **7.09**	− **0.42**	− **8.50**	− **7.48**	− **4.48**	− 29.61	
	*ccRCC 5*	− **2.63**	*1.92*	− **3.52**	*0.58*	− **3.53**	− **3.84**	− **5.20**	− 16.22	
	*ccRCC 6*	− **4.03**	− **6.87**	*2.14*	− **8.46**	*2.38*	*2.70*	− **0.17**	− 12.31	
	*ccRCC 7*	− **0.44**	− **2.23**	− **4.49**	− **4.30**	− **3.77**	− **3.17**	− **4.65**	− 23.05	
	*ccRCC 8*	− **3.02**	− **0.73**	− **0.56**	*0.52*	− **3.16**	− **0.68**	− **1.81**	− 9.44	
	*ccRCC 9*	− **1.44**	− **0.60**	− **1.60**	*1.30*	− **4.85**	− **3.28**	− **3.31**	− 13.78	
	*ccRCC 10*	− **3.35**	− **2.02**	− **3.62**	− **3.77**	− **3.22**	− **2.19**	*1.65*	− 16.52	
	*ccRCC 11*	*0.66*	− **5.13**	− **3.03**	− **3.25**	− **6.26**	− **7.35**	− **2.70**	− 27.06	
	*ccRCC 12*	− **2.49**	− **0.90**	− **7.25**	− **2.96**	− **6.54**	− **3.05**	*1.66*	− 21.53	
	*ccRCC 13*	− **2.87**	− **2.55**	*1.13*	− **7.10**	*4.33*	*1.86*	− **1.91**	− 7.11	
	*ccRCC 17*	*2.11*	− **3.03**	− **1.73**	− **2.63**	− **3.48**	− **2.59**	− **1.41**	− 12.76	
	*HS 1*	*4.48*	− **5.50**	*6.17*	− **2.72**	*2.04*	*2.36*	− **4.28**	2.55	
	*HS 2*	− **1.47**	− **1.64**	*0.55*	− **5.93**	*3.49*	*3.34*	− **6.32**	− 7.98	
	*HS 3*	− **0.92**	− **1.64**	*2.73*	− **3.73**	*3.47*	*1.97*	− **3.42**	− 1.54	
	*HS 4*	− **0.38**	− **4.34**	*2.31*	− **6.64**	*3.26*	*0.41*	− **0.28**	− 5.66	
	*HS 5*	− **0.23**	*0.91*	*0.47*	*3.34*	− **3.31**	*1.58*	− **2.93**	− 0.17	
	*HS 6*	− **0.04**	− **2.74**	*5.69*	− **4.92**	*6.52*	*5.03*	− **3.26**	6.28	
	*HS 7*	− **2.54**	*0.75*	− **1.57**	*1.46*	− **3.63**	*0.48*	− **0.66**	− 5.71	
	*HS 8*	*1.03*	*1.64*	− **3.12**	− **6.95**	*4.12*	*2.71*	*1.83*	1.26	
	*HS 9*	*1.21*	− **0.68**	− **0.80**	− **0.55**	− **2.28**	− **3.32**	*0.49*	− 5.93	
	*HS 10*	− **2.11**	*13.50*	− **13.64**	*1.77*	− **3.26**	*1.87*	− **0.32**	− 2.19	
	*HS 11*	− **0.54**	*8.67*	− **2.90**	− **1.49**	*5.91*	*5.55*	*16.48*	31.68	
	*HS 12*	− **0.20**	− **0.95**	− **5.65**	− **4.19**	− **3.76**	− **2.25**	− **0.56**	− 17.56	
	*HS 13*	*0.79*	− **2.01**	− **0.23**	− **4.14**	*0.55*	*3.70*	*5.57*	4.23	
	*HS 14*	*1.38*	− **1.37**	− **2.56**	− **0.25**	− **5.03**	*0.13*	*1.39*	− 6.31	

*Diseased values (value < − 7.10) and healthy values (value ≥ − 7.10).

**In italic the positive values which are in the healthy range; in bold the negative values which are in the diseased range.

**Figure 4:**
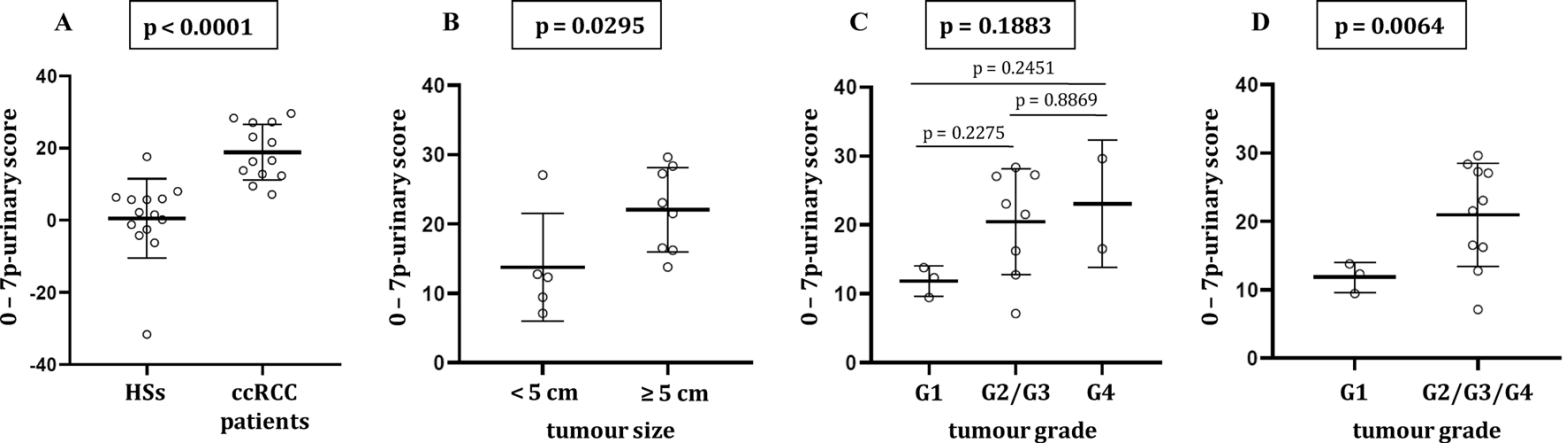
7p-urinary score of HSs and patients with ccRCC. (**A**) The 7p-urinary score mean of HSs is significantly different (unpaired t-test) from that of patients with ccRCC. (**B**) The 7p-urinary score mean of patients with ccRCC with a size smaller than 5 cm is significantly different (Mann–Whitney test) from that of patients with ccRCC size greater than 5 cm (tumour size is considered to be the largest diameter of the tumour as evaluated by CT scan). (**C**) The 7p-urinary score mean of patients with grade 1 (G1) ccRCC is not significantly different (ordinary one-way ANOVA—Tukey) from that of patients with ccRCC with grade 2/3 (G2/G3, same group) and grade 4 (G4). (**D**) The 7p-urinary score mean of patients with grade 1 ccRCC is significantly different (unpaired t-test) from that of patients with ccRCC with grade 2/3/4 (G2/G3/G4, same group).

The mean 7p-urinary score of ccRCC patients was significantly different from that of HSs (Fig. 4A). Interestingly, the mean 7p-urinary score was different in patients with tumour smaller and bigger than 5 cm (Fig. 4B), suggesting that the 7p-urinary score depends on the tumour size. Moreover, the mean 7p-urinary score appears different in patients with different grades of malignancy (Fig. 4C), but the difference was not significant, likely due to the small number of patients. When comparing the mean 7p-urinary score of patients with grade 1 tumours with the patients with grade 2–4 tumours (Fig. 4D), the mean 7p-urinary score was about two folds and the difference was significant. Supplementary Figure S8 compares the 7p-urinary scores of HSs with those of patients with ccRCC grouped considering tumour size (Supplementary Figure S8A) and grading (Supplementary Figure S8B).

In conclusion, the combination of urinary parameters based on miRNA presence gives a score (7p-urinary score) that has good sensitivity and specificity in evaluating the presence of ccRCC in a subject. The 7p-urinary score depends on the tumour size and tumour grade.

## Discussion

We investigated the possibility of diagnosing ccRCC through the evaluation of urinary miRNAs. We started with the analysis of data banks and its validation and then designed an algorithm to determine which miRNA is likely to be present in the urine at high levels in the presence of ccRCC. Amplification of chosen urinary miRNAs (miR-122, miR-1271, and miR-15b) and three internal controls allowed us to establish laboratory parameters (7p-urinary score) for discriminating patients with ccRCC from HSs with very high sensitivity and specificity.

Chow et al.^24^ have first described the overexpression of miR-122 in ccRCC specimens, and several studies have reported the effects of miR-122 on ccRCC proliferation and invasion^25,26^. The overexpression level of miR-122 found in this study was similar to that demonstrated in other studies^14,24^. Surprisingly, miR-122 was found to be underexpressed in the serum of patients with ccRCC compared with HSs^27^. In contrast, we found significantly higher amounts of miR-122 in the urine of patients with ccRCC than in that of HSs. This is the first report demonstrating a higher amount of miR-122 in the urine of patients with ccRCC.

In our study, miR-1271 was not overexpressed in ccRCC specimens but was increased in the urine of patients with ccRCC. There have been no reports of overexpression of miR-1271 in the urine of patients with ccRCC, but it has been reported that the expression levels of miR-1271 are 3.3-folds higher in ccRCC than in adjacent non-cancerous kidney tissue and 2.5-folds higher in the serum of patients with ccRCC than in that of HSs^28^. When evaluating the tissue expression of miR-1271, we used RNU6-2 as the reference gene, whose expression has been demonstrated to increase in some cancers^29^. Therefore, the low (and not significant) overexpression values of miR-1271 in ccRCC may have been due to increased expression of RNU6-2 in the tumour. The use of RNU6-2 as the only reference gene in tissue analysis is a limit of the study.

The results that we obtained studying miR-15b are quite interesting from a biological point of view. The expression of miR-15b is much higher in ccRCC than in healthy kidney specimens (17 folds, linear scale). However, we found that miR-15b expression is slightly higher in ccRCC specimens than in adjacent non-cancerous kidney tissues (2.6 folds, linear scale). The discrepancy may be due to interpatient variability or the different technique used to evaluate miRNA expression (microarray vs. RT-qPCR). However, the latter hypothesis appears unlikely, considering that data obtained with miR-122 were almost identical (53.5 vs. 51.0 folds, linear scale). Therefore, it appears more likely a biological reason specifically concerning miR-15b, such as, for example, the tendency of ccRCC-expressed miR-15b to diffuse and remain in healthy kidney tissue. Even more interesting is the lack of difference between the urinary level of miR-15b in ccRCC patients and HSs suggesting that miR-15b does not tend to move from ccRCC to the urine.

**Figure 5:**
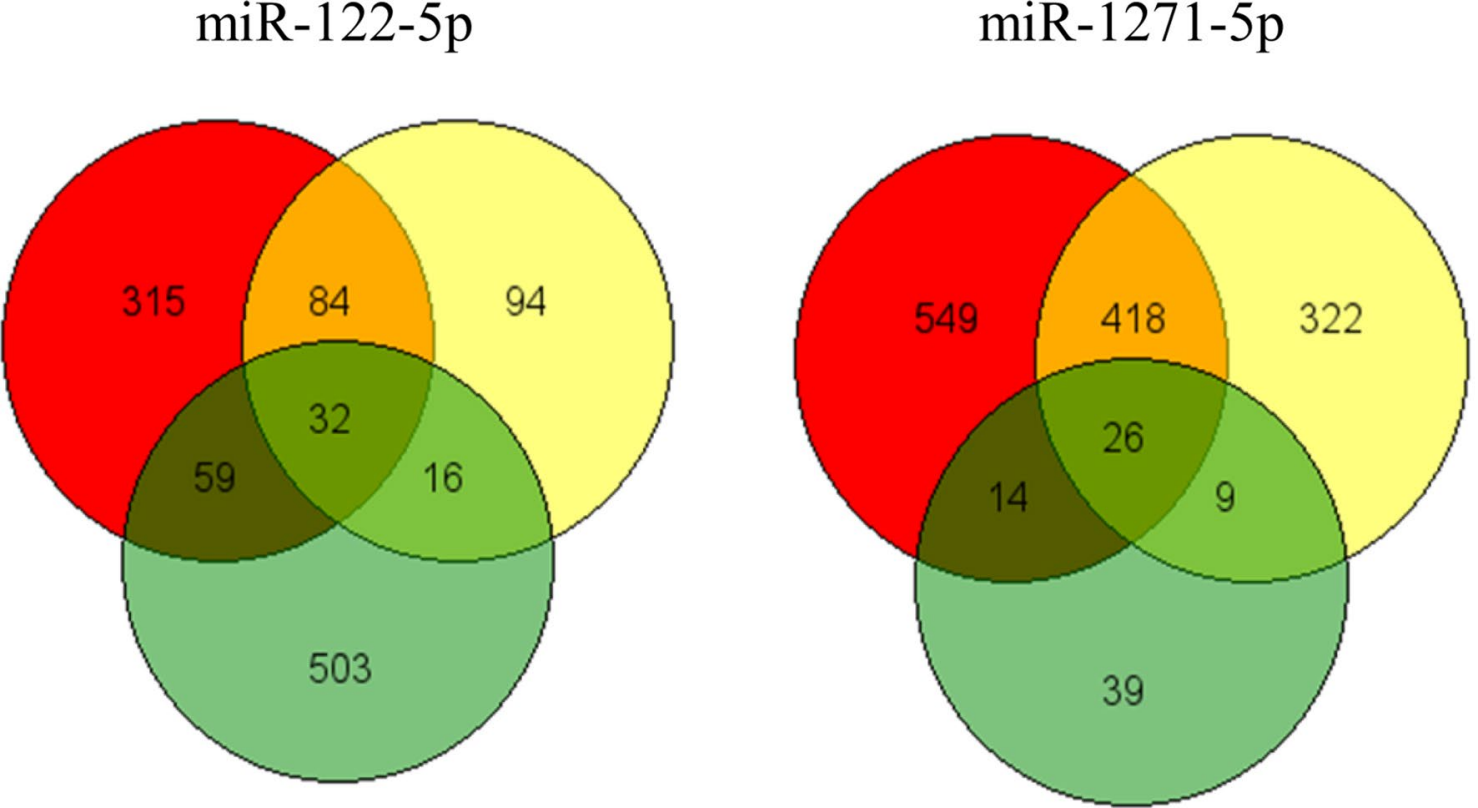
Selection of miRNA targets. The Venn diagrams represent the number of possible miRNA target genes as predicted by miRDB (red), TargetScan (yellow), and miRTarBase (green) tools. In particular, the number of target genes in each intersection and unique to a tool is represented. The genes selected by all the tools are considered to be the most likely miRNA target genes. The Venn diagram was drawn using the online software found at the following link http://bioinformatics.psb.ugent.be/webtools/Venn/.

To further confirm that miR-122 and miR-1271 play a role in ccRCC, we evaluated their potential gene targets (Fig. 5 and Supplementary Table S6). We are aware that identifying the miRNA targets is not an easy task and the effects of miRNAs on their potential targets must be confirmed by empirical data on the specific cells on which it is supposed they have effects. However, to find genes potentially dysregulated in the development and progression of the cancers among the miRNA targets may be an interesting starting point and may suggest which is the functional meaning of miRNA overexpression on tumours. We found some genes which may be targets of miR-122 and miR-1271 (Supplementary Table S7). In particular, Forkhead Box P1 (FOXP1), a member of the FOX family of transcription factors that have a critical role in the pathogenesis of cancer^30^, is among the targets of miR-122. In ccRCC, FOXP1 expression correlates inversely with tumour grade, and a loss of expression of FOXP1 is associated with a higher expression of ki-67, a relevant index of tumour proliferation^31^. This indicates that the potential miR-122-dependent decreased expression of FOXP1 favors ccRCC growth and is associated with a higher grade of malignancy. Moreover, one of the targets of the miR-1271 is the Regulator of G-protein signalling 2 (RGS2). RGS2 is a member of the RGS protein family that functions as a GTPase-activating protein Gα subunits of G-proteins^32^. RGS2 dysregulation is implicated in the development of solid tumours^33^. In particular, the expression of this gene has been shown downregulated in breast cancer^34^ and during the progression of ovarian^35^ and prostate cancer^36^.Therefore, the analysis of the miRNA target genes suggests that the miRNA selected by data bank analysis are miRNAs potentially involved in the development and progression of the cancers and specifically of ccRCC, as demonstrated by two studies on miR-122, demonstrating that its expression promotes malignant phenotypes and invasion of ccRCC^25,26^.

The idea of testing miRNAs in the urine for ccRCC diagnosis is not new. Von Brandenstein et al.^17^ and Mytsyk et al.^18^ found high levels of miR-15a in the urine of patients with RCC. Li et al.^19^ found that urinary levels of miR-210 were significantly higher in patients with ccRCC than in HSs, with sensitivity, specificity, and AUC equal to 58%, 80%, and 0.76, respectively. Similar data were obtained by Fedorko et al.^20^, who analyzed the urinary levels of let-7a and obtained an AUC of 0.83. Because of overlap in the miRNA values in the urine of the patients with ccRCC and HSs, the authors concluded that the data were not sufficient for the clinical application of let-7a as the only biomarker^20^. In our study, miR-122 and miR-1271 showed a sensitivity, specificity, and AUC similar to those obtained in the above-mentioned studies when they evaluated miRNAs different from miR-122 and miR-1271. Meanwhile, the combination of seven parameters (7p-urinary score) resulted in a 100% sensitivity, an 86% specificity, and an AUC equal to 0.96, demonstrating that the combination of more parameters increases the predictive value of the method. The seminal paper by Ramachandran et al. evaluated urinary miRNA expression in acute kidney injury and found four miRNAs capable of significantly differentiating patients with acute kidney injury from HSs^15^. However, in their study also the best AUC was obtained by combining the four miRNAs. For this reason, we plan to test even more urinary miRNAs to increase the number of parameters and further improve the specificity of the test.

When evaluating miRNA expression by cells, the use of internal control is useful to normalize the miRNA expression level based on (a) the number of cells; (b) the amount of extracted RNA; (c) RNA quality; and (d) the quality of the reactions (reverse transcription and PCR preamplification and amplification). When an established amount of urine is used to test the presence of a miRNA, normalization is less useful. Indeed, the eventual difference associated with (a) and (b) may be determined by the disease and should not be normalized. The RNA quality (c) is an issue, but the use of a stabilizer soon after urine collection reduces the likelihood of RNA degradation. For these reasons, some studies did not use any internal control when evaluating miRNA in the urine^19^. However, the lack of miRNA amplification in a urine sample may mean low quality of the starting RNA or failure of the reactions if not validated by the amplification of internal controls. The most frequently used internal control when evaluating the miRNA expression by cells is RNU6-2^37,38^. However, since RNU6-2 is not a miRNA and its release appears unlikely, it may be inappropriate to use RNU6-2 for normalization in liquid biopsy, where released miRNAs are investigated. The internal controls used in our study may be useful for the issue (c) (i.e., RNA quality) and issue (d) (e.g. reaction quality). Indeed, we used miR-16 to these aims, considering the data for urine samples reliable only when miR-16 showed Ct < 35. However, when we tried to use the internal control for normalization, we observed an unexpected finding. Normalization of miR-122 and miR-1271 with miR-16 slightly decreased the discriminating power of urinary miRNAs and normalization of miR-122, miR-1271, and miR-15b with cel-miR-39 and miRTC, increased the discriminating power but in an unexpected way (i.e. showing a decreased ΔCt value in patients with ccRCC). Therefore, miR-16 was used to normalize the expression of miR-122 and miR-1271 (parameters #4 and #7), showing that HSs had a mean ΔCt value higher than patients. On the contrary, cel-miR-39 was used to normalize the expression of miR-15b (parameter #12) and miRTC was used to normalize the expression of miR-122, miR-1271, and miR-15b (parameters #5, #8, and #11) showing that HSs had a mean ΔCt value lower than patients. In conclusion, our method uses both non-normalized Ct values (parameter #2) and normalized ΔCt values (the other six parameters) and the Ct of all miRNAs (both test and controls) are used to calculate the 7p-urinary score.

Our study may open a debate on the reliability of miRNA internal controls in the urine. As a first internal control, we tested the presence of miR-16. Although there was the possibility that in the urine of patients with ccRCC there was a different level of miR-16 as compared to that detected in the urine of HSs due to direct or indirect effects of cancer, we found no differences in the mean Ct of miR-16 in the urine of patients and HSs (about 1 Ct, p = 0.33; Supplementary Table S4). As a second and third internal controls, we used cel-miR-39 and miRTC. These miRNAs are not endogenous miRNAs but are added to the samples during laboratory procedures. In particular, cel-miR-39 is added before RNA extraction to evaluate the yield of RNA extraction and the subsequent reactions and miRTC is added before reverse transcriptase reaction and evaluate the yield of this reaction and PCR. In theory, in one laboratory the mean Ct of both cel-miR-39 and miRTC are similar in different groups of subjects, considering that reagents belong to the same batches (we performed RNA extraction, RT and PCR reactions coupling urine samples of patients and HSs), the device is always the same and people experimenting are the same. On the contrary, our results concerning amplification of cel-miR-39 suggest that there is a tendency to obtain more RNA or RNA of higher quality from the urine of the patients than from the urine of the HSs, even if the difference between the mean Cts is not significant (about 4 Cts, p = 0.06, Supplementary Table S4). To find more RNA in the urine of the patients may not be a laboratory artifact and may indicate that more cells and/or more miRNAs are present in the urine of the patients. However, this should not change the amount of purified cel-miR-39 unless we hypothesize the presence of factors favoring RNA purification or RT-PCR yield in the urine of patients or the presence of an endogenous miRNA similar to cel-miR-39 and overexpressed by cancer. Finally, it is even more surprising the difference between mean Cts of miRTC in the urine of patients and HSs (about 6 Cts, p = 0.0005; Supplementary Table S4). Reminding that miRTC is present in the RT mix and that we performed in pair urine of patients and HSs, it is unlikely to attribute the difference to laboratory artifacts. Instead, it may be thought that the urine of the patients contains factor(s) favoring the RT-PCR yield of miRTC, whose sequence is unknown, or contains an endogenous miRNA similar to miRTC and overexpressed by cancer. If the hypothesis will be confirmed, miRTC may represent a parameter indicating the presence of cancer, as suggested by Supplementary Figure S5. In conclusion, the three internal controls gave completely different results (the difference between the mean Cts of HSs and patients is equal to about 1—but not significant, 4—near to significance, and 6—highly significant) so that the idea of internal control and normalization appears not applicable to this context. For example, if we considered the presence of miR-122 in the urine of patients as compared to HSs, it appears overexpressed if considered without normalization or normalized by miR-16, expressed at similar levels if normalized by cel-miR-39, and expressed at lower levels if normalized by miRTC. Admitting that miR-16 is not expressed differently in the urine of patients as compared to the urine of HSs (true internal control), normalization with this miRNA appears the most reliable. However, we aimed to set up a method to apply in the clinic; therefore, following such unexpected results, we decided to consider Ct values of each miRNA independently from other considerations.

The specificity we obtained combining seven parameters was lower than that described by Mytsyk testing urinary for miR-15a alone, with a sensitivity and specificity equal to 100% and 98%, respectively^18^. However, the urinary data by Mytsyk et al. were normalized using RNU6-2 as an internal control that, in our opinion, is inappropriate, as discussed above. Indeed, when we tested the presence of RNU6-2 in the urine, about half of the urine samples from HSs did not show a Ct < 35 (not shown) and we believe it is risky to normalize miRNAs with a transcript present in the urine at very low levels and with high inter-subject variability, particularly in HSs. Moreover, miR-15a is present at high levels in the blood of patients with acute kidney injury^39^, suggesting that miR-15a may be present at high levels in the urine of patients without cancer but with non-cancerous diseases of the kidney, potentially increasing the false positive rate of the test. Our study suggests that evaluating the amplification of some miRNAs instead of that of one miRNA represents an advantage in patients in which alteration of the urinary presence of just one miRNA occurs for reasons that are not evident in clinics. Therefore, in our opinion, the 7p-urinary score may give more reliable results.

Despite it is known that cells deriving from kidney and excretory routes may be present in the urine, urine samples were not centrifuged before RNA extraction, for two reasons: (1) extracted miRNAs may derive from both urine and cells in the urine (including ccRCC cells), potentially favoring the presence of differences between ccRCC patients and HSs; (2) we used a protocol as easier and rapid as possible, to favor reproducibility of the method.

In this study, we compared the data for patients with ccRCC with those for the age- and sex-matched HSs and demonstrated that parameters derived from the evaluation of urinary miRNAs discriminated HSs from patients with ccRCC. The absolute value of the 7p-urinary score increases with the increase of tumour size and grade (Fig. 4).

The main limitations of our study are small numbers of ccRCC cases and controls and the absence of an independent validation set. Therefore, we considered this study a pilot study, showing the diagnostic potential of a combination of parameters based on the urinary concentrations of miRNAs and evaluation of internal controls; however, further independent studies are needed to confirm our results. Moreover, our method is potentially at high risk of false-positive results in subjects with urinary infection and inflammation and was not tested in these patients.

In conclusion, the combined use of urinary miR-122, miR-1271, miR-15b, and internal controls allows the diagnosis of ccRCC with high sensitivity and specificity. However, we performed an ad-hoc combination of the signals and, reasonably, sensitivity and specificity are affected by overfitting. Consequently, the performance of the present method (e.g. the AUC value) may be over-estimated. Therefore, our findings must be confirmed by studying an independent data set including more HSs and more patients. The method presented here is based on the miRNAs selected by using bioinformatic data and our algorithm. In the next future, we plan to test other urinary miRNAs together with the miRNAs tested here, to increase the AUC and specificity values of the 7p-urinary score by including more parameters. Moreover, it is necessary to test the sensitivity and specificity of the improved score in a sufficient number of patients with small cancer mass. If specificity would result sufficiently high, the improved method might have a predictivity sufficiently high to test the population at risk with a sufficiently low cost/benefit ratio.

## Materials and methods

### Subjects

For the discovery cohort, seventeen paired samples of fresh kidney cancer tissues and adjacent non-cancerous kidney tissue, as well as 13 urine samples, were obtained from patients affected by ccRCC, who underwent nephrectomy from May 2018 to March 2019. All the diagnoses were histologically verified. The study criteria included patients with ccRCC older than 30 years of age and excluded patients with urinary infections, renal lithiasis, and other neoplastic forms. Control urine samples were obtained from 14 sex- and age-matched HSs, without urinary infections, renal lithiasis, and different neoplastic types. The baseline renal function was evaluated based on serum creatinine and estimated glomerular filtration rate. Comorbidity profiles were classified according to the Charlson Comorbidity Index^40^. The demographic characteristics, smoking status, and renal function of the enrolled patients with ccRCC and HSs are reported in Supplementary Table S8. Of note, when the manuscript was in revision (more than one year after urine collection), HS #11 has been diagnosed a Hodgkin’s lymphoma but was not excluded from the HS group.

No patient with kidney cancer had any previous history of RCC. The mean clinical size of tumours was 6.36 cm (range: 2.1–14.0 cm). The R.E.N.A.L. nephrometry was also evaluated^41^. The clinicopathological data of ccRCC tumours are reported in Supplementary Table S9.

All interventions were carried out at the Urology Clinic of the S.M.M. Hospital, University of Perugia, Italy. Partial nephrectomy was performed in five of the 17 patients (29%). The collection of patient samples has been performed according to national legislation concerning ethical requirements. All methods were carried out following relevant guidelines as well as national and international laws and policies.

The CEAS Ethics Committee of Umbria Region approved the study (CEAS Register n° 3193/18), and written informed consent was obtained from the patients with ccRCC and HSs. All samples were evaluated anonymously.

### Data bank analysis

Expression data of 340 miRNAs from 166 arrays of healthy and ccRCC kidney samples [104, 9, 21 arrays from healthy human kidney, bladder, and prostate samples, respectively (control groups), and 32 arrays from ccRCC kidney samples (ccRCC group)], generated using the Affymetrix miRNA 1.0 HS platform, were downloaded in November 2018 through the Genevestigator suite (Nebion AG, Zurich, Switzerland)^23^. Gene expression data were obtained from datasets that are publicly available from Gene Expression Omnibus^42^ and the European Bioinformatics Institute^43^.

The microarray data are normalized by Genevestigator suite at two levels: Robust Multi-array Average (RMA) within experiments (through the Bioconductor package “affy” and a customized version of the package “affyExtensions”) and trimmed mean adjustment to a target for normalization between datasets determined by calculating the mean of all the expression values in an experiment (across all samples) after excluding the top and the bottom 5%. Normalization makes array data comparable, despite having been obtained in different laboratories^44,45^.

We included in the control group the arrays on samples that (1) were taken from HSs, and (2) were not subjected to experimental treatments. We included in the ccRCC group the arrays on samples that (1) were not subjected to experimental treatments, and (2) were not obtained by laser capture microdissection. The lists of the miRNAs and samples are reported in Supplementary Tables S10 and S11, respectively.

### Study of the potential gene targets of miRNAs

TargetScan v. 7.2^46^, TarBase v.8^47^, and miRDB v.6.0^48^ were used for the in silico prediction of genes targeted by miRNAs.

### Amplification of miRNAs from tissue by RT-qPCR

After nephrectomies, tissue samples were immersed in RNAlater (Thermo-Fisher Scientific, Waltham, Massachusetts, USA) and stored in the Oncological Urology Biobank at − 80 °C. ccRCC tissues were homogenized with Tissue Lyser (Qiagen, Hilden, Germany) in 600 µl of Lysis-Buffer, and RNeasy Mini Kit (Qiagen) was used for total RNA extraction. RNA concentration and purity were evaluated using Qubit4 Fluorometer and Nanodrop 2000c (Thermo-Fisher). Total RNA was reverse transcribed using miScriptII RT (Qiagen).

Expression of the test miRNAs and internal control (RNU6-2) (Supplementary Table S12) was evaluated by RT-qPCR in triplicate, with a no-template negative control, using a Rotor-GeneQ cycler (Qiagen) and the miScript SYBR Green PCR kit (Qiagen); the reaction conditions were 95 °C for 15 min, 45 cycles at 94 °C for 15 s, 55 °C for 30 s and 70 °C for 30 s. Samples were analyzed using the 2^−ΔΔCt^ method^49^. The Ct values of the tested miRNAs were considered reliable when the RNU6-2 Ct was less than 30. Raw data from RT-qPCR and data elaborated from RT-qPCR are shown in Supplementary Tables S13 and S14, respectively.

### Amplification of miRNAs from urine

Urine samples were taken in the afternoon from both patients and HSs. Specifically, urine samples from patients were collected after hospital admission (about 2 PM), the day before the surgery. Urine was stabilized within 4 h using a urine preservative (Norgen Biotek, Thorold, ON, Canada) and stored at 4 °C. Urine samples were not centrifuged before RNA extraction.

Total RNA was extracted from 200 μL of urine using the miRNeasy micro kit (Qiagen) after the addition of 5.6 × 10^8^ copies of the *Caenorhabditis elegans* miR-39 spike-in control (cel-miR-39; Qiagen). The RNA quality and concentration were evaluated using a NanoDrop 2000c (Thermo-Fisher). Urinary RNA was reverse transcribed using the miScriptII RT kit (Qiagen) containing miRTC, a proprietary synthetic RNA (Qiagen). Preamplification was performed using the miScriptPreAMP PCR kit (Qiagen). The preamplification cycling conditions were as follows: 95 °C for 15 min, followed by 12 cycles at 94 °C for 30 s and 60 °C for 3 min. Preamplified cDNA was diluted 20-fold. For each sample, amplification of three internal controls was performed: miR-16 (endogenous miRNA), cel-miR-39 (spike-in control, for determination of miRNA recovery from urinary samples), and miRTC (for assessment of reverse transcription efficiency) (Supplementary Table S12). Expression of the test miRNAs and internal controls was evaluated by RT-qPCR in triplicate, with a no-template negative control, using an ABI7300 cycler (Thermo-Fisher) and the miScriptSYBR Green PCR kit (Qiagen). The cycling conditions were as follows: 95 °C for 15 min, followed by 38 cycles at 94 °C for 15 s, 60 °C for 30 s, and 70 °C for 34 s. The miRNA amount was calculated based on the relative concentration of the target in the reaction using the cycle threshold (Ct) and the ΔCt method when specified. Reactions with melting curves different from expected were considered ‘no amplification’. Raw data from RT-qPCR are shown in Supplementary Tables S15 and S13, concerning the presence of urinary miRNA in HSs and patients with ccRCC, respectively.

### Statistical analysis

Kolmogorov–Smirnov (KS) normality test was performed on data from each group before statistical evaluation. To evaluate the differences between two groups, P values were calculated using an unpaired t-test (data bank and urine sample data) and a paired t-test (ccRCC and adjacent non-cancerous kidney tissue specimen data) when the KS normality test was passed, and Mann–Whitney test (data bank and urine sample data) and a Wilcoxon matched-pairs signed-rank test (ccRCC and corresponding adjacent non-cancerous kidney tissue specimen data) when KS normality test failed. To evaluate the differences between the three groups, P values were calculated using the ordinary one-way ANOVA (Tukey) test when the KS normality test was passed and the Kruskal–Wallis (Dunn) test when the KS normality test failed. Pearson correlation when the KS normality test was passed and Spearman correlation when the KS normality test failed were used to evaluate the correlations between parameters. All statistical analyses, including Receiver Operating Characteristic (ROC) curves, were performed using Prism 8.0.1 (GraphPad Software, San Diego, CA, USA).

## Supplementary information

Supplementary information.

Supplementary information.

Supplementary information.

Supplementary information.

Supplementary information.
