# Supplementary material for: Detection of urinary miRNAs for diagnosis of clear cell renal cell carcinoma
Source: Sci Rep. 2020 Dec 4;10:21290. doi: 10.1038/s41598-020-77774-9 (PMC7718885; doi:10.1038/s41598-020-77774-9)
Supplement: Supplementary file 1 — Supplementary information. [file 41598_2020_77774_MOESM1_ESM.pdf]

**Supplementary Figure**

# Detection of urinary miRNAs for diagnosis of clear cell renal cell carcinoma

**Giovanni Cochetti[1], Luigi Cari[2], Giuseppe Nocentini[2]*, Vincenza Maulà[1], Chiara Suvieri[1], Rosy Cagnani[1], Jacopo Adolfo Rossi de Vermandois[1], Ettore Mearini[1]**

[1] Urology Clinic, Department of Medicine and Surgery, University of Perugia, Perugia, Italy
[2] Pharmacology Section, Department of Medicine and Surgery, University of Perugia, Perugia, Italy

***Correspondence: giuseppe.nocentini@unipg.it; Tel.: +39-075-5858-126 (G.N.)

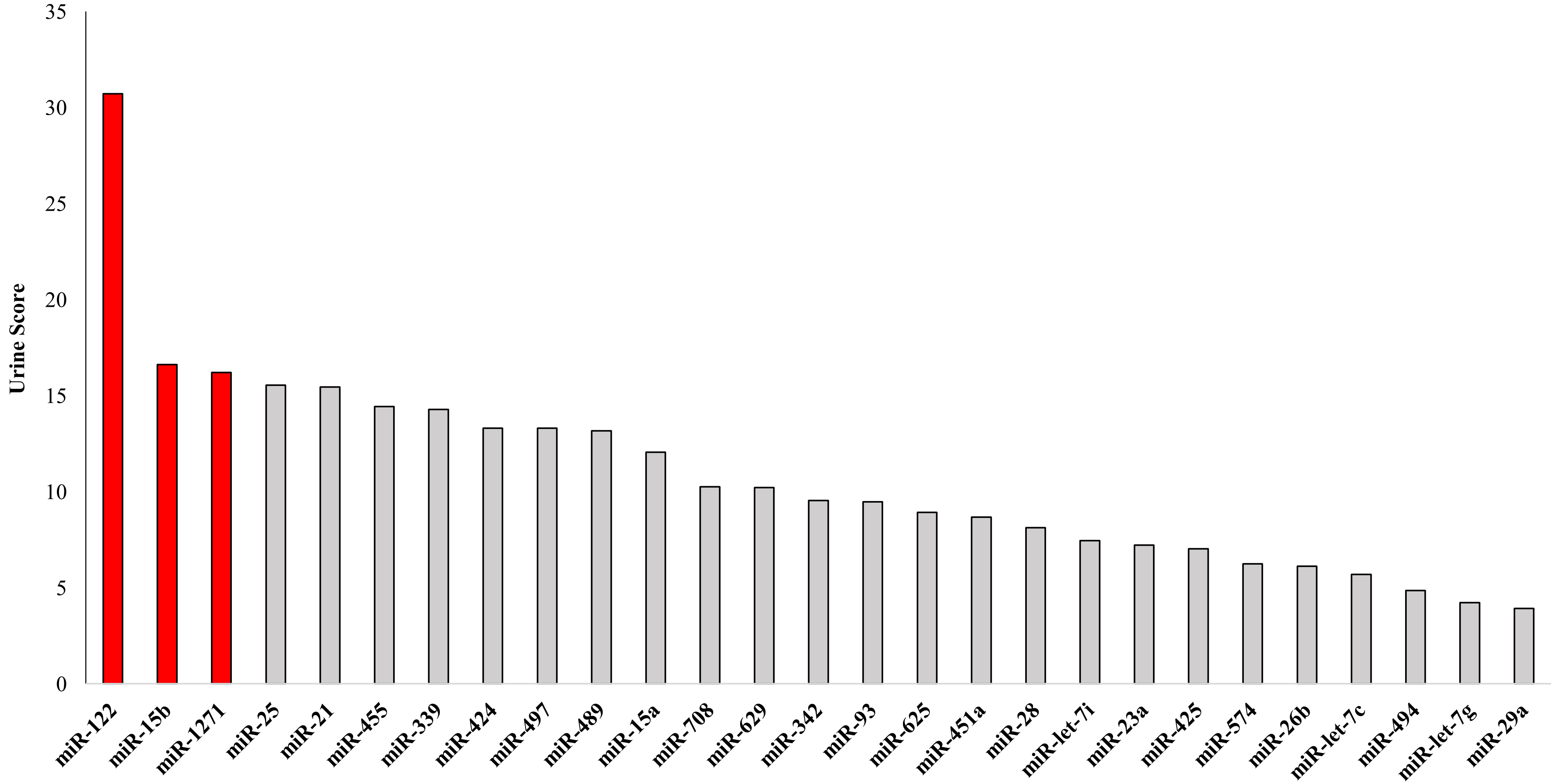


**Supplementary Figure S1. Urinary scores of miRNAs.** The miRNAs overexpressed at least threefold by ccRCC and their scores as derived by the algorithm. Scores represent the likelihood miRNAs are good urinary markers.

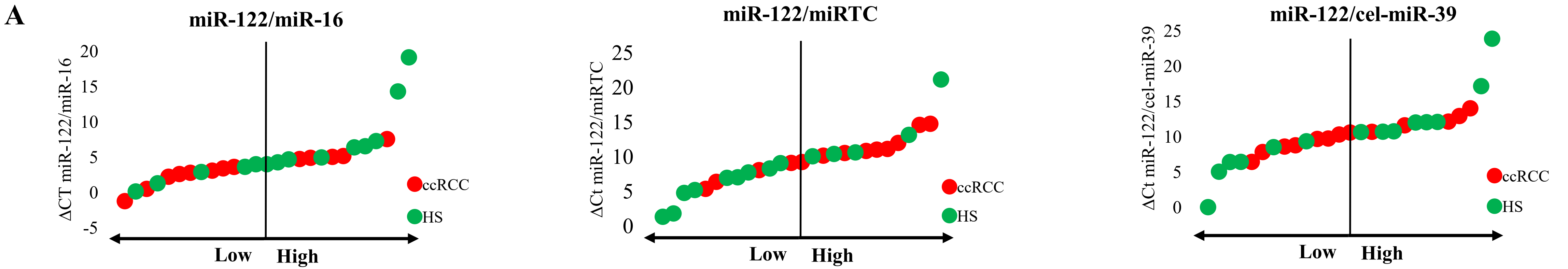


| | % Subject with ΔCt lower than median ΔCt | % Subject with ΔCt higher than median ΔCt |
|---|---|---|
| HSs | 38% | 62% |
| ccRCC | 62% | 38% |

| | % Subject with ΔCt lower than median ΔCt | % Subject with ΔCt higher than median ΔCt |
|---|---|---|
| HSs | 64% | 36% |
| ccRCC | 33% | 67% |

| | % Subject with ΔCt lower than median ΔCt | % Subject with ΔCt higher than median ΔCt |
|---|---|---|
| HSs | 43% | 57% |
| ccRCC | 58% | 42% |

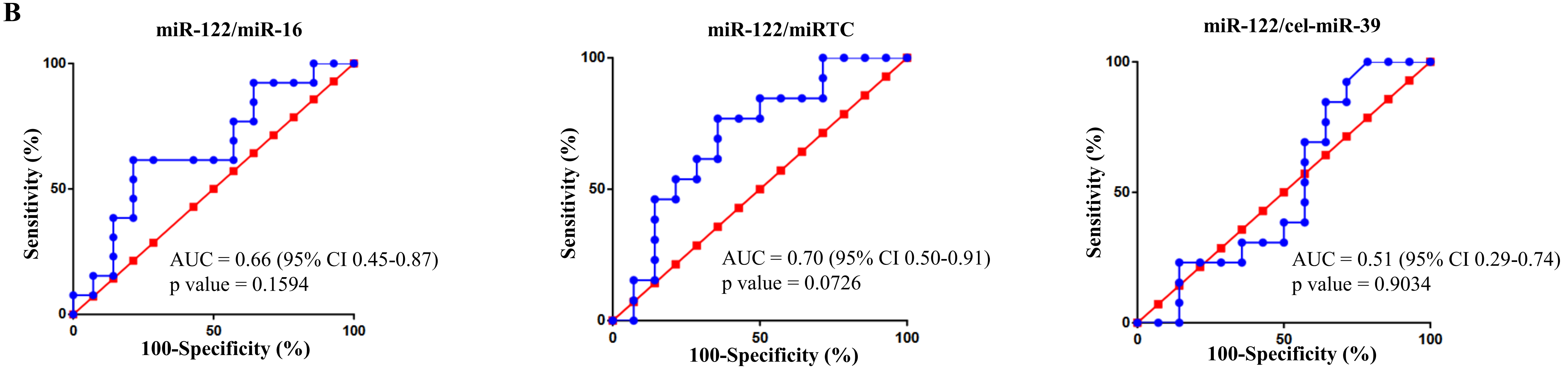


**Supplementary Figure S2. Expression levels of urinary miR-122 normalized by urinary internal controls and ROC curves. A**, ΔCt value of miR-122/miR-16, miR-122/miRTC, and miR-122/cel-miR-39 in the urine of HSs (green dots) and patients with ccRCC (red dots); in the Tables, the percentage of HSs and ccRCC patients with ΔCt value lower and higher than the median ΔCt is reported. Median ΔCt were equal to 3.97, 9.22, and 10.57 for miR-122/miR-16, miR-122/miRTC, and miR-122/cel-miR-39, respectively. **B**, ROC curve of miR-122/miR-16, miR-122/miRTC, and miR-122/cel-miR-39. Both panels **A** and **B** show that normalization by internal controls led to results different from each other and from those shown in Figure 2 in which expression levels of not-normalized urinary miR-122 are shown.

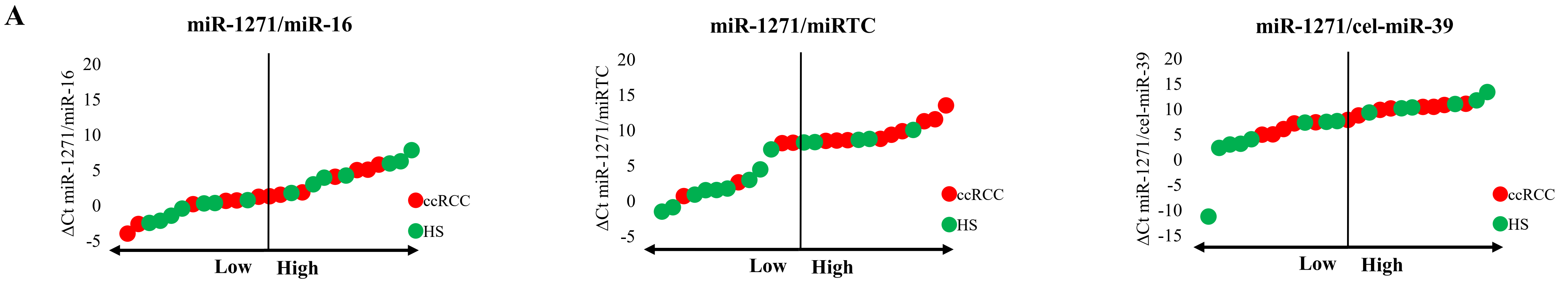


| | % Subject with ΔCt lower than median ΔCt | % Subject with ΔCt higher than median ΔCt |
|---|---|---|
| HSs | 50% | 50% |
| ccRCC | 50% | 50% |

| | % Subject with ΔCt lower than median ΔCt | % Subject with ΔCt higher than median ΔCt |
|---|---|---|
| HSs | 69% | 31% |
| ccRCC | 31% | 69% |

| | % Subject with ΔCt lower than median ΔCt | % Subject with ΔCt higher than median ΔCt |
|---|---|---|
| HSs | 57% | 43% |
| ccRCC | 42% | 58% |

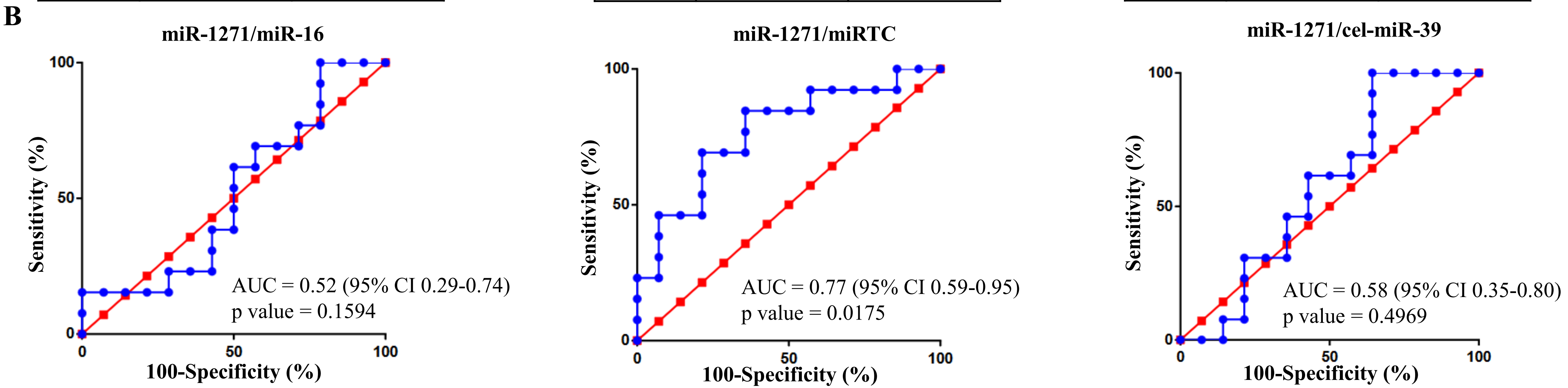


**Supplementary Figure S3. Expression levels of urinary miR-1271 normalized by urinary internal controls and ROC curves. A**, ΔCt value of miR-1271/miR-16, miR-1271/miRTC, and miR-1271/cel-miR-39 in the urine of HSs (green dots) and patients with ccRCC (red dots); in the Tables, the percentage of HSs and ccRCC patients with ΔCt value lower and higher than the median ΔCt is reported. Median ΔCt were equal to 1.28, 8.25, and 7.83 for miR-1271/miR-16, miR-1271/miRTC, and miR-1271/cel-miR-39, respectively. **B**, ROC curve of miR-1271/miR-16, miR-1271/miRTC, and miR-1271/cel-miR-39. Both panels **A** and **B** show that normalization by internal controls led to results different from each other and from those shown in Figure 2 in which expression levels of not-normalized urinary miR-1271 are shown.

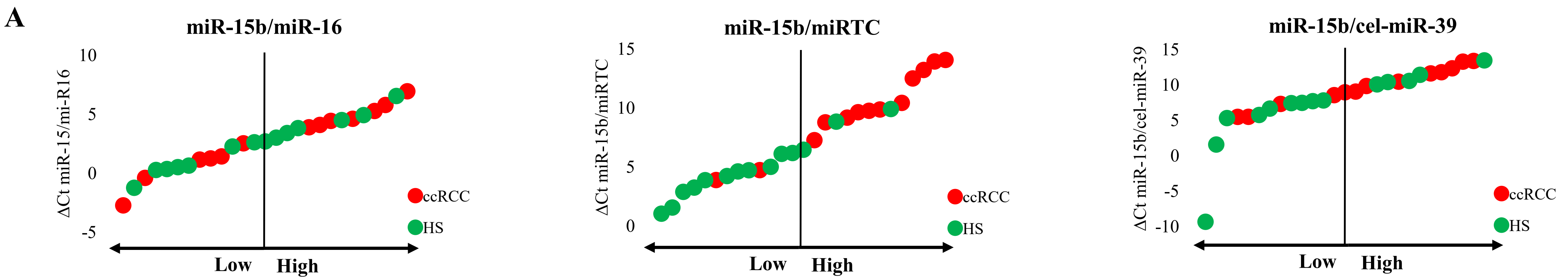


**miR-15b/miR-16**

| | % Subject with ΔCt lower than median ΔCt | % Subject with ΔCt higher than median ΔCt |
|---|---|---|
| HSs | 54% | 46% |
| ccRCC | 46% | 54% |

**miR-15b/miRTC**

| | % Subject with ΔCt lower than median ΔCt | % Subject with ΔCt higher than median ΔCt |
|---|---|---|
| HSs | 85% | 15% |
| ccRCC | 15% | 85% |

**miR-15b/cel-miR-39**

| | % Subject with ΔCt lower than median ΔCt | % Subject with ΔCt higher than median ΔCt |
|---|---|---|
| HSs | 64% | 36% |
| ccRCC | 33% | 67% |

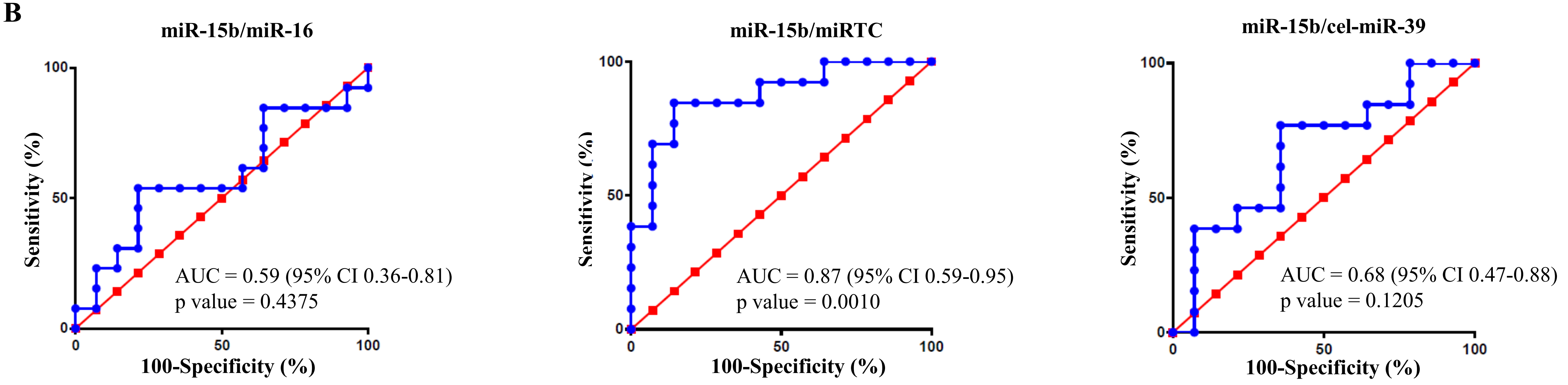


**Supplementary Figure S4. Expression levels of urinary miR-15b normalized by urinary internal controls and ROC curves. A**, ΔCt value of miR-15b/miR-16, miR-15b/miRTC, and miR-15b/cel-miR-39 in the urine of HSs (green dots) and patients with ccRCC (red dots); in the Tables, the percentage of HSs and ccRCC patients with ΔCt value lower and higher than the median ΔCt is reported. Median ΔCt were equal to 2.67, 6.43, and 8.91 for miR-15b/miR-16, miR-15b/miRTC, and miR-15b/cel-miR-39, respectively. **B**, ROC curve of miR-15b/miR-16, miR-15b/miRTC, and miR-15b/cel-miR-39. Both panels **A** and **B** show that normalization by internal controls led to results different from each other and from those shown in Figure 2 in which expression levels of not-normalized urinary miR-15b are shown.

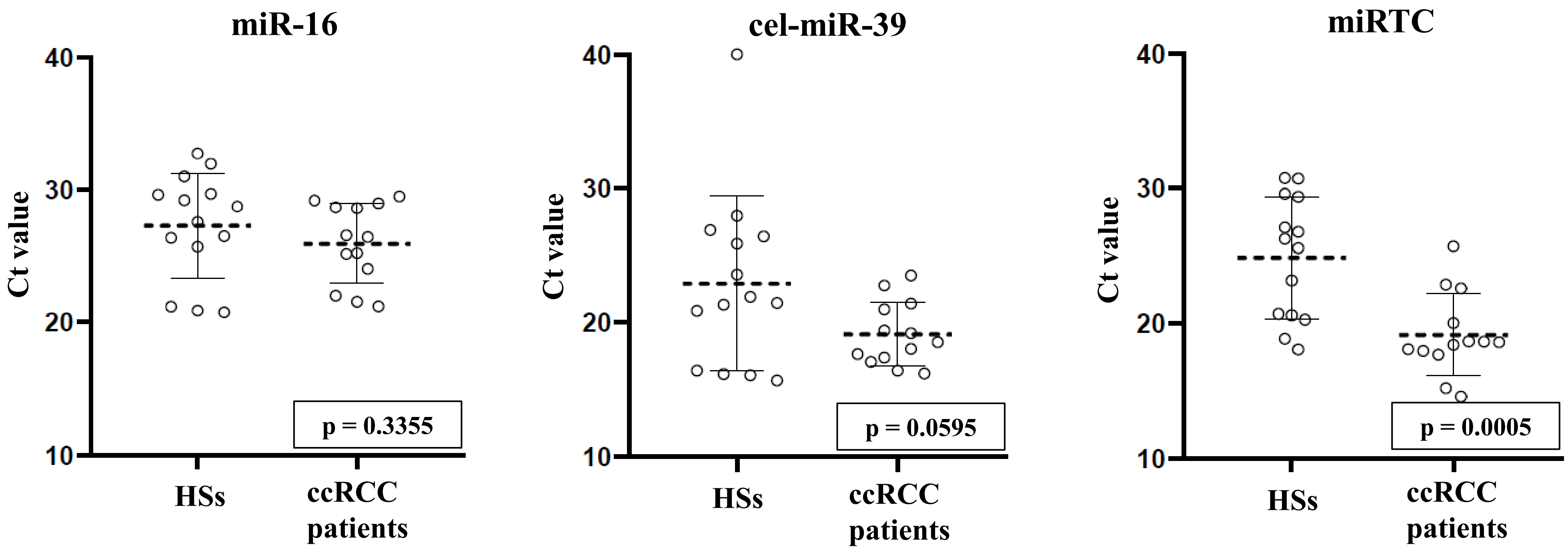


**Supplementary Figure S5. Mean Ct values of internal controls in urine.** **A**, The mean miR-16 Ct value of HSs is not significantly different from that of patients with ccRCC, using the unpaired t-test. **B**, The mean cel-miR-39 Ct value of HSs is not significantly different from that of patients with ccRCC, using the unpaired t-test. **C**, The mean miRTC Ct value of HSs is significantly different from that of patients with ccRCC, using the Mann-Whitney test.

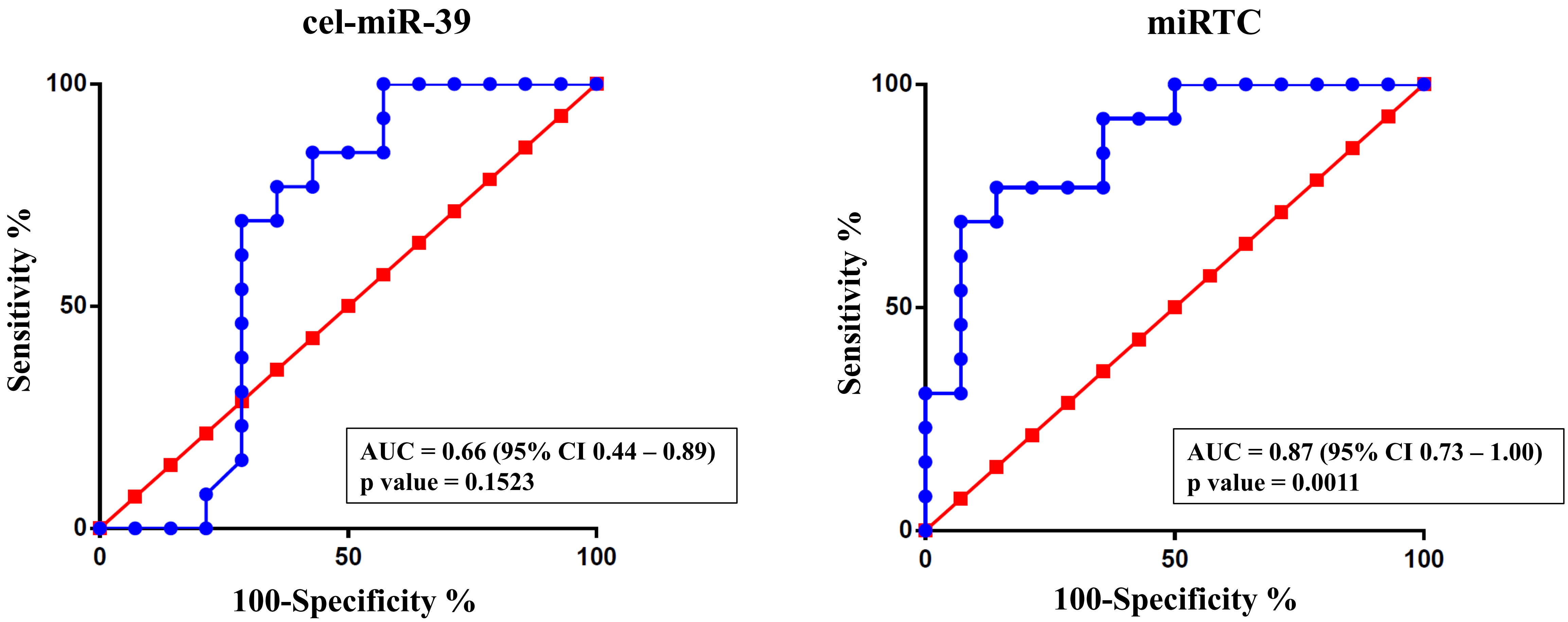


**Supplementary Figure S6. ROC curves of internal controls.** ROC curves of cel-miR-39 (left), and miRTC (right) are reported.

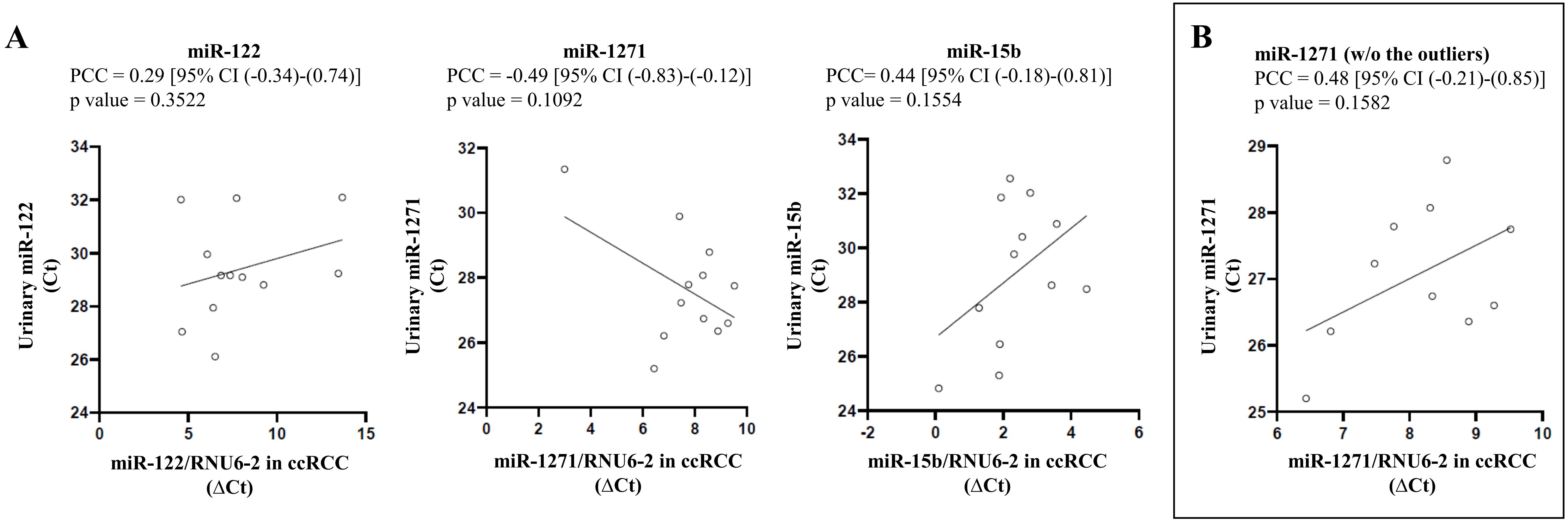


**Supplementary Figure S7. Correlation between miRNA expression by cancer specimens and their presence in the corresponding urine. A**, Correlation between miR-122, miR-1271 and miR-15b expression by RNU6-2-normalized ccRCC specimens and presence of not-normalized miRNA in the corresponding urine sample of ccRCC patients. Pearson correlation coefficient (PCC) and p value are reported. **B**, Correlation between RNU6-2-normalized miR-1271 by ccRCC specimens and not-normalized miR-1271 presence in the corresponding urine sample, without considering patients #11 and #17, representing outliers**.** Pearson correlation coefficient (PCC) and p value are reported.

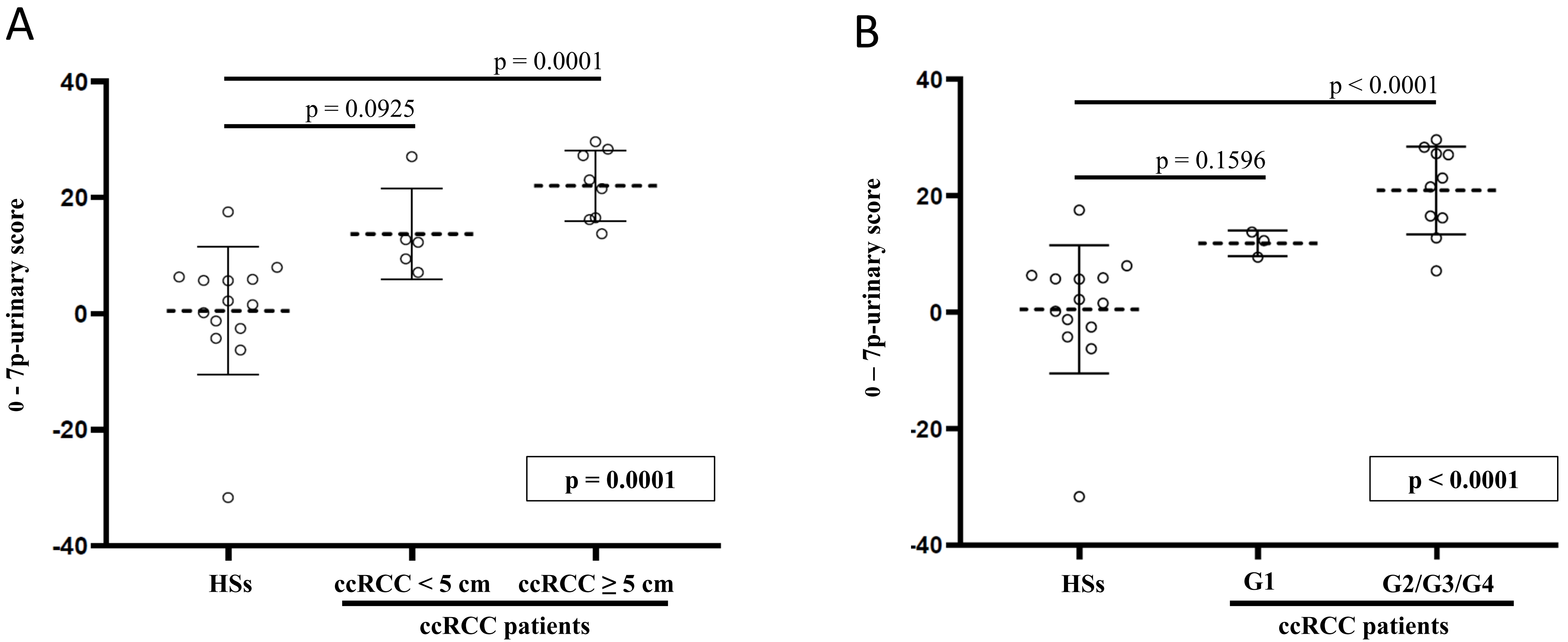


**Supplementary Figure S8. 7p-urinary score of healthy subjects (HSs) and patients with ccRCC. A**, The 7p-urinary score of HSs is compared with that of patients divided on the basis of the tumour size considering as size the largest diameter of the tumour evaluated by CT scan at time of the diagnosis; p values were calculated using Kruskal-Wallis (Dunn) test. **B**, The 7p-urinary score of HSs is compared with that of patients divided on the basis of tumour grade (G); p values were calculated using ordinary one-way ANOVA (Tukey).
