# Supplementary material for: Detection of urinary miRNAs for diagnosis of clear cell renal cell carcinoma
Source: Sci Rep. 2020 Dec 4;10:21290. doi: 10.1038/s41598-020-77774-9 (PMC7718885; doi:10.1038/s41598-020-77774-9)
Supplement: Supplementary file 2 — Supplementary information. [file 41598_2020_77774_MOESM2_ESM.pdf]

Supplementary Tables

Detection of urinary miRNAs for diagnosis of clear cell renal cell carcinoma

Giovanni Cochetti[1], Luigi Cari[2], Giuseppe Nocentini[2]*, Vincenza Maulà[1], Chiara Suvieri[1], Rosy Cagnani[1], Jacopo Adolfo Rossi de Vermandois[1], Ettore Mearini[1]

[1] Urology Clinic, Department of Medicine and Surgery, University of Perugia, Perugia, Italy
[2] Pharmacology Section, Department of Medicine and Surgery, University of Perugia, Perugia, Italy

*Correspondence: giuseppe.nocentini@unipg.it; Tel.: +39-075-5858-126 (G.N.)

Supplementary Table S1. miRNA expression by ccRCC as resulting from data bank analysis. (A) miRNAs overexpressed at least 1.73 Log_2 folds (3.3 folds on linear scale) and (B) miRNAs underexpressed at least 0.77 Log_2 folds (1.7 folds on linear scale) in ccRCC samples as compared to kidney samples from healthy subjects.

A

miRNA	ccRCC folds overexpression (Log_2)	p value (unpaired t-test)
**miR-122**	5.67	<0.0001
**miR-15b**	4.10	<0.0001
**miR-21**	4.02	<0.0001
**miR-25**	3.62	<0.0001
**miR-494**	3.59	<0.0001
**miR-497**	3.52	<0.0001
**miR-451a**	3.48	<0.0001
**miR-1271**	3.46	<0.0001
**miR-15a**	3.16	<0.0001
**miR-425**	2.75	<0.0001
**miR-339**	2.59	<0.0001
**miR-342**	2.46	<0.0001
**miR-629**	2.39	<0.0001
**miR-489**	2.07	0.0006
**miR-424**	2.06	0.0001
**miR-625**	2.04	<0.0001
**miR-26b**	2.03	<0.0001
**miR-28**	2.03	<0.0001
**miR-let-7**i	2.00	0.0017
**miR-let-7g**	1.97	0.0014
**miR-574**	1.89	<0.0001
**miR-455**	1.85	<0.0001
**miR-let-7c**	1.85	0.0092
**miR-93**	1.82	<0.0001
**miR-23a**	1.82	<0.0001
**miR-29a**	1.81	0.0033
**miR-708**	1.76	0.0003

B

miRNA	ccRCC folds underexpression (Log_2)	P value (unpaired t-test)
**miR-369**	0.77	<0.0001
**miR-488**	0.77	<0.0001
**miR-607**	0.78	<0.0001
**miR-576**	0.80	0.0428
**miR-570**	0.81	0.0003
**miR-942**	0.81	<0.0001
**miR-606**	0.82	0.0001
**miR-578**	0.82	<0.0001
**miR-208a**	0.85	0.0001
**miR-1255a**	0.85	<0.0001
**miR-585**	0.87	0.0014
**miR-1827**	0.87	<0.0001
**miR-379**	0.89	0.0005
**miR-506**	0.89	<0.0001
**miR-548e**	0.90	<0.0001
**miR-508**	0.92	0.0006
**miR-1231**	0.94	0.0006
**miR-1281**	0.94	0.0121
**miR-603**	1.05	0.0005
**miR-1228**	1.07	0.0159
**miR--874**	1.13	0.0022
**miR-548j**	1.15	<0.0001
**miR-370**	1.17	0.0004
**miR-663a**	1.29	0.0289
**miR-92b**	1.34	0.0004
**miR-510**	1.35	<0.0001
**miR-933**	1.63	<0.0001
**miR-572**	1.68	0.0037
**miR-638**	1.73	<0.0001
**miR-1260a**	1.83	<0.0001

Supplementary Table S2. miRNA expression by ccRCC as resulting from experimental data from our laboratory. Expression of the miRNAs in ccRCC as compared to the adjacent non-cancerous kidney tissue sample in each patient. Values (expressed as Log_2) indicating overexpression by ccRCC samples are in red and values (expressed as Log_2) indicating underexpression by ccRCC samples are in grey.

Patient #	miR-122	miR-1271	miR-15b	miR-625	miR-629	miR-93	miR-1260a	miR-369
1	7.54	-0.20	3.28	2.09	1.27	1.00	0.82	-7.29
2	-2.90	0.93	2.58	-1.08	-2.51	-0.90	1.09	5.01
3	6.64	1.07	2.33	0.78	2.13	0.91	1.46	-1.03
4	5.78	0.39	1.13	0.53	0.64	-1.61	-0.27	2.26
5	6.94	0.04	-0.10	-1.33	-2.65	-1.66	3.55	-4.50
7	3.70	0.88	0.60	0.02	0.57	1.34	-1.15	-1.14
8	9.52	2.49	2.60	0.20	1.90	-0.05	-0.26	-1.67
9	9.37	0.61	3.06	2.99	1.33	-0.14	1.09	1.43
12	4.53	-0.97	-0.76	3.40	0.35	-0.60	-0.81	-3.20
13	5.44	0.70	1.06	-0.20	0.9	0.96	-4.69	-1.97
14	8.18	1.44	-0.79	1.29	-0.98	-1.81	-1.44	-6.06
15	7.61	0.64	1.63	1.62	1.21	0.25	-0.74	1.24
16	6.03	-1.50	1.36	3.78	0.93	0.45	-2.19	1.48
17	2.00	-1.99	1.40	2.00	3.28	1.74	1.90	4.00

**Mean modulation**	5.74	0.32	1.38	1.15	0.60	-0.01	-0.12	-0.82
**Passed KS test ***	YES	NO	NO	YES	YES	NO	NO	YES
**p value**	<0.0001	0.3258	0.0031	0.0180	0.1993	0.9515	0.9878	0.4158
**% patients with ccRCC overexpressing miRNA**	93 %	71 %	79 %	79 %	79 %	50 %	43 %	43 %
**% patients with ccRCC underexpressing miRNA**	7 %	29 %	21 %	21 %	21 %	50 %	57 %	57 %
**SD**	3.24	1.18	1.32	1.59	1.65	1.17	2.01	3.64
**Min modulation**	-2.90	-1.99	-0.79	-1.33	-2.65	-1.81	-4.69	-7.29
**Max modulation**	9.52	2.49	3.28	3.78	3.28	1.74	3.55	5.01

* Kolmogorov-Smirnov (KS) test (a normality test)

Supplementary Table S3. Urine scores of the miRNAs overexpressed at least threefold in ccRCC and the values used by the algorithm to obtain the urine score. Values are from data bank analysis and are expressed as Log_2.

Factor *	3	-1.5	-1	1	1 **	SCORE		
**miRNA**	**ccRCC overexpression**	**SD Kidney**	**SD ccRCC**	**Kidney/ Bladder ratio**	**Kidney/ Prostate ratio**	**Female score**	**Male score**	**Total score**
**miR-122**	5.67	0.28	1.82	-0.48	2.12	14.3	16.4	**30.7**
**miR-15b**	4.10	1.67	0.44	-0.13	-1.87	9.2	7.4	**16.6**
**miR-1271**	3.46	1.10	0.76	0.44	-0.61	8.4	7.8	**16.2**
**miR-25**	3.62	1.64	0.68	0.80	-1.54	8.5	7.0	**15.5**
**miR-21**	4.02	1.90	0.84	0.15	-1.62	8.5	6.9	**15.4**
**miR-455**	1.85	1.22	0.83	3.73	1.16	6.6	7.8	**14.4**
**miR-339**	2.59	1.15	0.40	1.57	-0.14	7.2	7.1	**14.3**
**miR-424**	2.06	1.46	1.01	2.32	2.69	5.3	8.0	**13.3**
**miR-497**	3.52	1.64	0.68	1.11	-3.78	8.5	4.8	**13.3**
**miR-489**	2.07	1.40	2.01	2.66	3.65	4.8	8.4	**13.2**
**miR-15a**	3.16	1.43	0.42	0.95	-3.69	7.9	4.2	**12.1**
**miR-708**	1.76	1.39	1.13	2.05	2.02	4.1	6.1	**10.2**
**miR-629**	2.39	0.40	0.80	0.10	-1.52	5.9	4.3	**10.2**
**miR-342**	2.46	1.51	0.43	0.33	-0.52	5.0	4.5	**9.5**
**miR-93**	1.82	0.80	0.45	0.43	1.00	4.2	5.2	**9.5**
**miR-625**	2.04	0.55	0.74	-0.07	-0.02	4.5	4.4	**8.9**
**miR-451a**	3.48	1.06	2.33	-1.07	-2.22	5.4	3.2	**8.7**
**miR-28**	2.03	1.28	0.48	0.89	-1.03	4.6	3.5	**8.1**
**miR-let-7i**	2.00	2.09	0.42	1.59	-0.63	4.0	3.4	**7.5**
**miR-23a**	1.82	1.22	0.23	0.38	-0.33	3.8	3.4	**7.2**
**miR-425**	2.75	1.58	0.57	-0.84	-1.88	4.5	2.6	**7.0**
**miR-574**	1.89	1.36	0.47	0.73	-1.55	3.9	2.3	**6.2**
**miR-26b**	2.03	0.77	0.77	0.61	-3.46	4.8	1.3	**6.1**
**miR-let-7c**	1.85	2.40	0.30	1.23	-0.08	2.9	2.8	**5.7**
**miR-494**	3.59	2.38	0.88	-1.39	-5.00	4.9	-0.1	**4.9**
**miR-let-7g**	1.97	2.00	0.54	1.35	-3.22	3.7	0.5	**4.2**
**miR-29a**	1.81	2.05	0.32	1.46	-3.05	3.5	0.4	**3.9**

* Factor by which values were multiplied

** Only for men.

Supplementary Table S4. Presence of miRNAs in the urine. Ct of urinary miR-122, miR-1271, and miR-15b in ccRCC patients and HSs obtained from RT-qPCR and reported as Ct value. miR-16, miRTC, and cel-miR-39 were used as internal controls.

		miR-16 Ct	miRTC Ct	cel-miR-39 Ct	miR-122 Ct	miR-1271 Ct	miR-15b Ct
ccRCC	2	26.47	18.41	17.64	29.23	27.75	30.88
	3	26.60	18.66	18.51	28.80	27.23	31.85
	4	24.03	14.58	17.04	29.16	28.07	28.62
	5	21.56	18.08	16.18	29.09	26.60	28.48
	6	29.20	22.59	19.18	27.94	25.20	26.45
	7	28.63	20.03	18.01	32.01	28.79	29.76
	8	21.23	18.06	16.39	26.11	26.21	25.30
	9	22.03	17.95	17.38	27.04	27.79	27.79
	10	25.19	17.67	20.97	28.78	25.88	26.42
	11	28.68	18.64	22.75	29.16	29.89	32.55
	12	25.24	15.21	19.38	29.95	26.74	24.82
	13	29.00	25.70	21.39	32.06	26.36	30.40
	17	29.51	22.87	23.51	32.09	31.34	32.02
**Mean ccRCC**		**25.95**	**19.11**	**19.10**	**29.34**	**27.53**	**28.87**
**SD ccRCC**		**3.02**	**3.07**	**2.39**	**1.85**	**1.71**	**2.61**
HSs	1	31.97	30.76	23.58	32.08	33.71	34.96
	2	29.23	26.26	16.06	33.20	27.76	29.48
	3	27.58	26.79	20.86	31.55	28.31	31.38
	4	31.03	27.12	25.89	32.30	28.85	33.27
	5	21.20	20.70	15.65	27.72	29.00	25.68
	6	29.65	30.72	21.89	32.52	29.19	32.25
	7	20.77	18.07	16.39	27.13	26.69	24.15
	8	32.75	29.39	27.97	40.00	30.26	33.24
	9	26.53	23.17	26.44	31.46	30.44	33.05
	10	20.89	18.87	16.14	40.00	27.12	23.56
	11	25.72	29.61	40.00	40.00	28.69	30.62
	12	28.76	20.28	21.43	33.42	29.03	29.09
	13	29.70	25.58	26.91	33.30	30.02	28.44
	14	26.40	20.59	21.31	30.64	30.61	27.02
**Mean HSs**		**27.30**	**24.85**	**22.89**	**33.24**	**29.26**	**29.73**
**SD HSs**		**3.99**	**4.51**	**6.52**	**4.12**	**1.74**	**3.60**

	miR-16 Ct	miRTC Ct	cel-miR-39 Ct	miR-122 Ct	miR-1271 Ct	miR-15b Ct
**Mean ccRCC / Mean HSs**	**-1.35**	**-5.74**	**-3.79**	**-3.90**	**-1.74**	**-0.86**
**Passed KS test ***	**YES**	**NO**	**YES**	**NO**	**YES**	**YES**
**p value ****	**0.3355**	**0.0005**	**0.0595**	**0.0032**	**0.0150**	**0.4890**

* Kolmogorov-Smirnov (KS) test (a normality test)

** Difference between the mean Ct of urinary miRNAs in patients with ccRCC and HSs

Supplementary Table S5. Twelve parameters reported as Ct or ΔCt. ΔCt were calculated using Ct data reported in Supplementary Table S4.

Parameter		# 1	# 2	# 3	# 4	# 5	# 6	# 7	# 8	# 9	# 10	# 11	# 12
Parameter description		miR-122 Ct	miR-1271 Ct	miR-15b Ct	miR-122 / miR-16 ΔCt	miR-122 / miRTC ΔCt	miR-122 / cel-miR-39 ΔCt	miR-1271 / miR-16 ΔCt	miR-1271 / miRTC ΔCt	miR-1271 / cel-miR-39 ΔCt	miR-15b / miR-16 ΔCt	miR-15b / miRTC ΔCt	miR-15b / cel-miR-39 ΔCt
ccRCC	2	29.23	27.75	30.88	2.76	10.82	11.59	1.28	9.34	10.11	4.41	12.47	13.24
	3	28.80	27.23	31.85	2.20	10.14	10.29	0.63	8.57	8.72	5.25	13.19	13.34
	4	29.16	28.07	28.62	5.13	14.58	12.12	4.04	13.49	11.03	4.59	14.04	11.58
	5	29.09	26.60	28.48	7.53	11.01	12.91	5.04	8.52	10.42	6.92	10.4	12.30
	6	27.94	25.20	26.45	-1.26	5.35	8.76	-4.00	2.61	6.02	-2.75	3.86	7.27
	7	32.01	28.79	29.76	3.38	11.98	14.00	0.16	8.76	10.78	1.13	9.73	11.75
	8	26.11	26.21	25.30	4.88	8.05	9.72	4.98	8.15	9.82	4.07	7.24	8.91
	9	27.04	27.79	27.79	5.01	9.09	9.66	5.76	9.84	10.41	5.76	9.84	10.41
	10	28.78	25.88	26.42	3.59	11.11	7.81	0.69	8.21	4.91	1.23	8.75	5.45
	11	29.16	29.89	32.55	0.48	10.52	6.41	1.21	11.25	7.14	3.87	13.91	9.80
	12	29.95	26.74	24.82	4.71	14.74	10.57	1.50	11.53	7.36	-0.42	9.61	5.44
	13	32.06	26.36	30.40	3.06	6.36	10.67	-2.64	0.66	4.97	1.40	4.70	9.01
	17	32.09	31.34	32.02	2.58	9.22	8.58	1.83	8.47	7.83	2.51	9.15	8.51
**Mean ccRCC**		**29.34**	**27.53**	**28.87**	**3.39**	**10.23**	**10.24**	**1.58**	**8.42**	**8.43**	**2.92**	**9.76**	**9.77**
**SD ccRCC**		**1.85**	**1.71**	**2.61**	**2.23**	**2.74**	**2.10**	**2.89**	**3.42**	**2.21**	**2.72**	**3.21**	**2.67**
HSs	1	32.08	33.71	34.96	0.11	1.32	8.50	1.74	2.95	10.13	2.99	4.20	11.38
	2	33.20	27.76	29.48	3.97	6.94	17.14	-1.47	1.50	11.70	0.25	3.22	13.42
	3	31.55	28.31	31.38	3.97	4.76	10.69	0.73	1.52	7.45	3.80	4.59	10.52
	4	32.30	28.85	33.27	1.27	5.18	6.41	-2.18	1.73	2.96	2.24	6.15	7.38
	5	27.72	29.00	25.68	6.52	7.02	12.07	7.80	8.30	13.35	4.48	4.98	10.03
	6	32.52	29.19	32.25	2.87	1.80	10.63	-0.46	-1.53	7.30	2.60	1.53	10.36
	7	27.13	26.69	24.15	6.36	9.06	10.74	5.92	8.62	10.30	3.38	6.08	7.76
	8	40.00	30.26	33.24	7.25	10.61	12.03	-2.49	0.87	2.29	0.49	3.85	5.27
	9	31.46	30.44	33.05	4.93	8.29	5.02	3.91	7.27	4.00	6.52	9.88	6.61
	10	40.00	27.12	23.56	19.11	21.13	23.86	6.23	8.25	10.98	2.67	4.69	7.42
	11	40.00	28.69	30.62	14.28	10.39	0	2.97	-0.92	-11.31	4.90	1.01	-9.38
	12	33.42	29.03	29.09	4.66	13.14	11.99	0.27	8.75	7.60	0.33	8.81	7.66
	13	33.30	30.02	28.44	3.60	7.72	6.39	0.32	4.44	3.11	-1.26	2.86	1.53
	14	30.64	30.61	27.02	4.24	10.05	9.33	4.21	10.02	9.30	0.62	6.43	5.71
**Mean HSs**		**33.24**	**29.26**	**29.73**	**5.94**	**8.39**	**10.34**	**1.96**	**4.41**	**6.37**	**2.43**	**4.88**	**6.83**
**SD HSs**		**4.12**	**1.74**	**3.60**	**5.03**	**4.95**	**5.61**	**3.28**	**4.00**	**6.18**	**2.15**	**2.49**	**5.53**
**Mean ccRCC / Mean HSs**		-3.90	-1.74	-0.86	-2.55	1.84	-0.11	-0.39	4.00	2.06	0.49	4.88	2.94
**Passed KS test ***		NO	YES	YES	NO	YES	NO	YES	NO	YES	YES	YES	NO
**p value**		0.0032	0.0150	0.4890	0.1654	0.2478	0.9154	0.7470	0.0168	0.2682	0.6054	0.0002	0.1279
**Range of values shown by diseased patients**	**Mean - SD**	27.49	25.82	26.26	1.16	7.49	8.14	-1.31	4.99	6.22	0.20	6.56	7.10
	**Mean + SD**	31.19	29.23	31.48	5.61	12.97	12.34	4.46	11.84	10.63	5.64	12.97	12.44

* Kolmogorov-Smirnov (KS) test (a normality test)

Supplementary Table S6. Target genes of miR-122 and miR-1271. Target genes were identified by each of the three tools miRDB, TargetScan, and miRTarBase tools, as represented in Figure 5.

hsa-miR-122-5p	
**mRNA Target**	**Ensembl ID**
ADAM10	ENSG00000137845
ALDOA	ENSG00000149925
ANKRD13C	ENSG00000118454
CCNG1	ENSG00000113328
CLIC4	ENSG00000169504
CS	ENSG00000062485
CTDNEP1	ENSG00000175826
FOXK2	ENSG00000141568
FOXP1	ENSG00000114861
FUNDC2	ENSG00000165775
G6PC3	ENSG00000141349
H1F0	ENSG00000189060
HECTD3	ENSG00000126107
KDELC2	ENSG00000178202
NPEPPS	ENSG00000141279
NT5C3A	ENSG00000122643
P4HA1	ENSG00000122884
PDK4	ENSG00000004799
PEG10	ENSG00000242265
PIP4K2A	ENSG00000150867
PKM	ENSG00000067225
PRKRA	ENSG00000180228
PXMP4	ENSG00000101417
RBM47	ENSG00000163694
SLC52A2	ENSG00000185803
SLC7A1	ENSG00000139514
SMYD4	ENSG00000186532
SPRY2	ENSG00000136158
ST6GALNAC4	ENSG00000136840
SUCLA2	ENSG00000136143
TBC1D22B	ENSG00000065491
TFDP2	ENSG00000114126

hsa-miR-1271-5p	
**mRNA Target**	**Ensembl ID**
ACER2	ENSG00000177076
BRWD1	ENSG00000185658
CCNG1	ENSG00000113328
CSNK1D	ENSG00000141551
DDAH1	ENSG00000153904
EIF4EBP2	ENSG00000148730
EVI5	ENSG00000067208
FOXO1	ENSG00000150907
FOXQ1	ENSG00000164379
GABRB1	ENSG00000163288
GID4	ENSG00000141034
IGF1R	ENSG00000140443
MAP3K3	ENSG00000198909
MORF4L1	ENSG00000185787
NPTX1	ENSG00000171246
PFN1	ENSG00000108518
PPP1R11	ENSG00000204619
PPP1R12A	ENSG00000058272
RGS2	ENSG00000116741
SH3BGRL3	ENSG00000142669
SIN3B	ENSG00000127511
STK17B	ENSG00000081320
TMEM170B	ENSG00000205269
TRIM7	ENSG00000206557
USP5	ENSG00000111667
ZFP36L1	ENSG00000185650

Supplementary Table S7. Target genes of miR-122-5p, miR-1271-5p likely involved in the development and progression of cancers. Target genes were identified by each of the three tools miRDB, TargetScan, and miRTarBase tools, as represented in Figure 5 and selected among the genes shown in the Table S6.

**ID miRNA**	**mRNA target**	**Aliases Gene**	**Ensembl ID**
hsa-miR-122-5p	CCNG1<br>FOXP1<br>TFDP2	Cyclin G1<br>Forkhead Box P1<br>Transcription Factor Dp-2	ENSG00000113328<br>ENSG00000114861<br>ENSG00000114126
hsa-miR-1271-5p	SIN3B<br>MAP3K3<br>RGS2<br>IGF1R<br>CCNG1<br>CSNF1D	SIN3 Transcription Regulator Family Member B<br>Mitogen-Activated Protein Kinase Kinase Kinase 3<br>Regulator Of G Protein Signaling 2<br>Insulin Like Growth Factor 1 Receptor<br>Cyclin G1<br>Casein Kinase 1 Delta	ENSG00000127511<br>ENSG00000198909<br>ENSG00000116741<br>ENSG00000140443<br>ENSG00000113328<br>ENSG00000141551

Supplementary Table S8. Demographic characteristics, smoke status, and clinical features. Demographic characteristics, smoke status, renal function, and comorbidities of ccRCC patients and healthy subjects (HSs) from which presence of miRNAs in the urine was evaluated by qRT-PCR (discovery cohort). From the same patients, ccRCC specimen and adjacent non-cancerous kidney tissue was taken and analysed for miRNA expression.

	**ccRCC** (n: 17)	**HSs** (n: 14)
**Sex**		
Male	13	10
Female	4	4
**Age**		
Mean ± SD	63.71 ± 10.15	57.14 ± 8.34
Range	49-83	42-79
**Smoke**		
Smoker/Ex smoker	8	5
Non-smoker	9	9
**Serum creatinine (mL/dL)**		
Mean ± SD	1.00 ± 0.24	0.82 ± 0.16
**eGFR (MDRD equation, mL/min x 1.73 m^2)**		
Mean ± SD	79.98 ± 22.79	97.87 ± 18.03
**Charlson Comorbidity Index**		
Mean (range)	6 (2-9)	4 (0-7)

Supplementary Table S9. Clinicopathological data of ccRCC tumours of discovery cohort described in Table S8.

Fuhrman grade	
G1	4
G2	8
G3	3
G4	2
**T stage**	
T1	10
T2	0
T3	7
T4	0
**M stage**	
M0	13
M1	4
**N stage**	
N0	14
N1	1
Nx	2

Tumour mass weight (g)	
Mean	46.60
Range	11-127
**Tumour mass size (cm) ***	
Mean	6.36
Range	2.1-14.0
**R.E.N.A.L. score**	
Mean	9
Range	4-12
**Surgery type**	
Partial nephrectomy	5 (29.41%)
Total nephrectomy	12 (70.59%)

* Largest Diameter evaluated by CT scan

Supplementary Table S10. List of the miRNAs evaluated with the data bank analysis. miRNA were 340 in total.

miR-10a	miR-1260a	miR-151a	miR-345	miR-490	miR-554	miR-595	miR-634	miR-767
miR-10b	miR-1261	miR-15a	miR-346	miR-491	miR-555	miR-596	miR-635	miR-769
miR-1178	miR-1262	miR-15b	miR-361	miR-492	miR-556	miR-597	miR-636	miR-770
miR-1179	miR-1263	miR-17	miR-362	miR-493	miR-557	miR-598	miR-637	miR-802
miR-1180	miR-1265	miR-1825	miR-363	miR-494	miR-558	miR-599	miR-638	miR-873
miR-1181	miR-1266	miR-1827	miR-367	miR-495	miR-559	miR-600	miR-639	miR-874
miR-1182	miR-1267	miR-18b	miR-369	miR-496	miR-561	miR-601	miR-640	miR-875
miR-1183	miR-1268a	miR-202	miR-370	miR-497	miR-562	miR-602	miR-641	miR-876
miR-1197	miR-1269a	miR-208a	miR-373	miR-498	miR-563	miR-603	miR-642a	miR-877
miR-1200	miR-1271	miR-20b	miR-375	miR-500a	miR-564	miR-604	miR-643	miR-887
miR-1202	miR-1272	miR-21	miR-377	miR-501	miR-566	miR-605	miR-644a	miR-888
miR-1203	miR-1273a	miR-22	miR-378a	miR-502	miR-567	miR-606	miR-645	miR-889
miR-1204	miR-1275	miR-23a	miR-379	miR-503	miR-568	miR-607	miR-646	miR-890
miR-1205	miR-1276	miR-23b	miR-381	miR-504	miR-569	miR-608	miR-647	miR-920
miR-1207	miR-1277	miR-25	miR-382	miR-505	miR-570	miR-609	miR-648	miR-921
miR-1208	miR-1278	miR-26b	miR-383	miR-506	miR-571	miR-610	miR-649	miR-922
miR-122	miR-1281	miR-28	miR-409	miR-507	miR-572	miR-611	miR-650	miR-92b
miR-1226	miR-1282	miR-297	miR-410	miR-508	miR-573	miR-613	miR-651	miR-93
miR-1227	miR-1284	miR-298	miR-411	miR-510	miR-574	miR-614	miR-652	miR-933
miR-1228	miR-1286	miR-29a	miR-412	miR-513c	miR-575	miR-615	miR-653	miR-934
miR-1229	miR-1287	miR-300	miR-421	miR-525	miR-576	miR-616	miR-654	miR-935
miR-1231	miR-1288	miR-31	miR-423	miR-527	miR-577	miR-617	miR-655	miR-936
miR-1234	miR-1290	miR-32	miR-424	miR-532	miR-578	miR-618	miR-656	miR-937
miR-1236	miR-1292	miR-320a	miR-425	miR-539	miR-579	miR-619	miR-657	miR-938
miR-1237	miR-1293	miR-323a	miR-429	miR-541	miR-580	miR-620	miR-658	miR-939
miR-1238	miR-1294	miR-324	miR-431	miR-542	miR-581	miR-621	miR-659	miR-940
miR-1245a	miR-1295a	miR-325	miR-432	miR-543	miR-582	miR-622	miR-660	miR-942
miR-1247	miR-1296	miR-326	miR-433	miR-544a	miR-583	miR-623	miR-661	miR-943
miR-1249	miR-1298	miR-328	miR-448	miR-545	miR-584	miR-624	miR-662	miR-944
miR-1250	miR-1299	miR-330	miR-451a	miR-548e	miR-585	miR-625	miR-663a	miR-95
miR-1251	miR-1301	miR-331	miR-452	miR-548g	miR-586	miR-626	miR-671	miR-96
miR-1252	miR-1303	miR-335	miR-454	miR-548j	miR-587	miR-627	miR-675	miR-98
miR-1253	miR-1304	miR-337	miR-455	miR-548l	miR-588	miR-628	miR-708	miR-let-7c
miR-1254-1	miR-1305	miR-339	miR-483	miR-548n	miR-589	miR-629	miR-744	miR-let-7d
miR-1255a	miR-1306	miR-33a	miR-484	miR-548p	miR-590	miR-630	miR-758	miR-let-7g
miR-1256	miR-1307	miR-33b	miR-485	miR-549a	miR-591	miR-631	miR-760	miR-let-7i
miR-1257	miR-1323	miR-340	miR-488	miR-552	miR-592	miR-632	miR-765	
miR-1258	miR-1324	miR-342	miR-489	miR-553	miR-593	miR-633	miR-766	

Supplementary Table S11. List of the samples evaluated with the data bank analysis. Sample name and study code of the sample are reported.

Healthy samples (n=104)					
Study code	Sample name	Study code	Sample name	Study code	Sample name
**HS-01175**	con_kid_1-1	**HS-01366**	CI high_kid_2-1	**HS-01413**	ACR_kid_3-8
**HS-01175**	con_kid_1-2	**HS-01366**	CI high_kid_2-2	**HS-01413**	ACR_kid_3-9
**HS-01175**	con_kid_1-3	**HS-01366**	CI high_kid_2-3	**HS-01413**	ACR_kid_3-10
**HS-01175**	con_kid_1-4	**HS-01366**	CI high_kid_2-4	**HS-01413**	ACR_kid_3-11
**HS-01175**	con_kid_1-5	**HS-01366**	CI high_kid_2-5	**HS-01413**	ACR_kid_3-12
**HS-01175**	con_kid_1-6	**HS-01366**	CI high_kid_2-6	**HS-01413**	ACR_kid_3-13
**HS-01175**	con_kid_1-7	**HS-01366**	CI high_kid_2-7	**HS-01413**	ACR_kid_3-14
**HS-01175**	con_kid_1-8	**HS-01366**	CI high_kid_2-8	**HS-01413**	ACR_kid_3-15
**HS-01175**	con_kid_1-9	**HS-01366**	CI high_kid_2-9	**HS-01413**	ACR_kid_3-16
**HS-01175**	con_kid_1-10	**HS-01413**	con_kid_1-1	**HS-01413**	ACR_kid_3-17
**HS-01175**	con_kid_1-11	**HS-01413**	con_kid_1-2	**HS-01413**	ACR_kid_3-18
**HS-01175**	con_kid_1-12	**HS-01413**	con_kid_1-3	**HS-01413**	ACR_kid_3-19
**HS-01175**	con_kid_1-13	**HS-01413**	con_kid_1-4	**HS-01413**	ACR_kid_3-20
**HS-01175**	con_kid_1-14	**HS-01413**	con_kid_1-5	**HS-01413**	ACR_kid_3-21
**HS-01175**	con_kid_1-15	**HS-01413**	con_kid_1-6	**HS-01413**	ACR_kid_3-22
**HS-01175**	con_kid_1-16	**HS-01413**	con_kid_1-7	**HS-01413**	ACR_kid_3-23
**HS-01175**	con_kid_1-17	**HS-01413**	con_kid_1-8	**HS-01413**	ACR_kid_3-24
**HS-01222**	con_kid_1-1	**HS-01413**	con_kid_1-9	**HS-01413**	ACR_kid_3-25
**HS-01222**	con_kid_1-2	**HS-01413**	ABMR_kid_2-1	**HS-01413**	ACR_kid_3-26
**HS-01222**	con_kid_1-3	**HS-01413**	ABMR_kid_2-2	**HS-01413**	ACR_kid_3-27
**HS-01222**	con_kid_1-4	**HS-01413**	ABMR_kid_2-3	**HS-01413**	DGF_kid_4-1
**HS-01222**	con_kid_1-5	**HS-01413**	ABMR_kid_2-4	**HS-01413**	DGF_kid_4-2
**HS-01222**	con_kid_1-6	**HS-01413**	ABMR_kid_2-5	**HS-01413**	DGF_kid_4-3
**HS-01222**	con_kid_1-7	**HS-01413**	ABMR_kid_2-6	**HS-01413**	DGF_kid_4-4
**HS-01222**	con_kid_1-8	**HS-01413**	ABMR_kid_2-7	**HS-01413**	DGF_kid_4-5
**HS-01222**	con_kid_1-9	**HS-01413**	ABMR_kid_2-8	**HS-01413**	DGF_kid_4-6
**HS-01222**	con_kid_1-10	**HS-01413**	ABMR_kid_2-9	**HS-01413**	DGF_kid_4-7
**HS-01222**	con_kid_1-11	**HS-01413**	ABMR_kid_2-10	**HS-01413**	DGF_kid_4-8
**HS-01222**	con_kid_1-12	**HS-01413**	ACR_kid_3-1	**HS-01413**	DGF_kid_4-9
**HS-01366**	CI low_kid_1-1	**HS-01413**	ACR_kid_3-2	**HS-01413**	DGF_kid_4-10
**HS-01366**	CI low_kid_1-2	**HS-01413**	ACR_kid_3-3	**HS-01413**	DGF_kid_4-11
**HS-01366**	CI low_kid_1-3	**HS-01413**	ACR_kid_3-4	**HS-01413**	DGF_kid_4-12
**HS-01366**	CI low_kid_1-4	**HS-01413**	ACR_kid_3-5	**HS-01413**	DGF_kid_4-13
**HS-01366**	CI low_kid_1-5	**HS-01413**	ACR_kid_3-6	**HS-01413**	DGF_kid_4-14
**HS-01366**	CI low_kid_1-6	**HS-01413**	ACR_kid_3-7		

ccRCC samples (n=32)	
Study code	Sample name
**HS-01175**	prim_kid rcc_4-1
**HS-01175**	prim_kid rcc_4-2
**HS-01175**	prim_kid rcc_4-3
**HS-01175**	prim_kid rcc_4-4
**HS-01175**	prim_kid rcc_4-5
**HS-01222**	prim_kid cca_2-1
**HS-01222**	prim_kid cca_2-2
**HS-01222**	prim_kid cca_2-3
**HS-01222**	prim_kid cca_2-4
**HS-01222**	prim_kid cca_2-5
**HS-01222**	prim_kid cca_2-6
**HS-01222**	prim_kid cca_2-7
**HS-01222**	prim_kid cca_2-8
**HS-01222**	prim_kid cca_2-9
**HS-01222**	prim_kid cca_2-10
**HS-01222**	prim_kid cca_2-11
**HS-01222**	prim_kid cca_2-12
**HS-01222**	prim_kid cca_2-13
**HS-01175**	prim_kid paca_2-1
**HS-01175**	prim_kid paca_2-2
**HS-01175**	prim_kid paca_2-3
**HS-01175**	prim_kid paca_2-4
**HS-01175**	prim_kid paca_2-5
**HS-01175**	prim_kid paca_2-6
**HS-01175**	prim_kid paca_2-7
**HS-01175**	prim_kid paca_3-1
**HS-01175**	prim_kid paca_3-2
**HS-01175**	prim_kid paca_3-3
**HS-01175**	prim_kid paca_3-4
**HS-01175**	prim_kid paca_3-5
**HS-01175**	prim_kid paca_3-6
**HS-01175**	prim_kid paca_3-7

Supplementary Table S12. Primers. (A) Primers used to amplify mature miRNAs and **(B)** internal controls. Catalog number refers to Qiagen products.

A

miRNA	Catalog number
hsa-miR-122-5p	MS00003416
hsa-miR-1271-5p	MS00014399
hsa-miR-15b-5p	MS00008792
hsa-miR-625-5p	MS00033894
hsa-miR-629-5p	MS00010395
hsa-miR-93-5p	MS00003346
hsa-miR-1260a	MS00014329
hsa-miR-369-3p	MS00006853

B

Internal control	Catalog number
hsa-RNU6-2	MS00033740
hsa-miR-16	MS00031493
miRTC	MS00000001
cel-miR-39	MS00019789
